# Supplementary material for: The strigolactone receptor D14 targets SMAX1 for degradation in response to GR24 treatment and osmotic stress
Source: Plant Commun. 2022 Jan 31;3(2):100303. doi: 10.1016/j.xplc.2022.100303 (PMC9073322; doi:10.1016/j.xplc.2022.100303)
Supplement: Document S1. Supplemental information, Supplemental Figures 1–11, and Supplemental Table 1 [file mmc1.pdf]

Plant Communications, Volume *3*

Supplemental information

The strigolactone receptor D14 targets SMAX1 for degradation in response to GR24 treatment and osmotic stress

Qingtian Li, Elena Sánchez Martín-Fontecha, Aashima Khosla, Alexandra R.F. White, Sunhyun Chang, Pilar Cubas, and David C. Nelson

Supplemental Information

The strigolactone receptor D14 targets SMAX1 for degradation in response to GR24 treatment and osmotic stress

Qingtian Li[1], Elena Sánchez Martín-Fontecha[2], Aashima Khosla[1], Alexandra R.F. White[1], Sunhyun Chang[1], Pilar Cubas[2], David C. Nelson[1C]

[1]Department of Botany and Plant Sciences, University of California, Riverside, CA 92521, United States of America

[2]Plant Molecular Genetics Department, Centro Nacional de Biotecnología/CSIC, Campus Universidad Autónoma de Madrid, Madrid, Spain

[C]Corresponding author, david.nelson@ucr.edu

This PDF file includes:

Supplemental information text

Supplemental figures 1 to 11

Supplemental table 1

Supplemental Information text

Extended Description of Materials and Methods

Plant growth conditions

Plants were grown under white light (MaxLite LED T8 4000K, ~110 µmol m^{-2} s^{-1}) with LD photoperiod (16 h light/8 h dark) at 21°C. Soil was supplemented with Gnatrol WDG and Marathon (imidacloprid). *Agrobacterium tumefaciens* (GV3101 pMP90)–mediated transformation of *Arabidopsis* was performed using the floral dip method as described previously (Clough and Bent, 1998). All characterized transgenic lines were homozygous.

Chemical compounds

KAR_1, KAR_2 and *rac*-GR24 were synthesized as previously reported (Goddard-Borger et al., 2007). $GR24^{5DS}$ and $GR24^{\textit{ent}\text{-}5DS}$ enantiomers were purified from *rac*-GR24 by chiral-phase HPLC as described (Scaffidi et al., 2014). 10 mM or 50 mM stocks were prepared in acetone and stored at -20°C, and freshly diluted in aqueous solutions before use.

Hypocotyl assay

Hypocotyl growth under red light was performed as described previously, but in a HiPoint DCI-700 LED Z4 growth chamber (Nelson et al., 2011). Surface-sterilized seeds were plated on 0.5x Murashige-Skoog (MS) media with 1 µM KAR_2, 1 µM *rac*-GR24, 0.5 µM $GR24^{5DS}$, 0.5 µM $GR24^{\textit{ent}\text{-}5DS}$ or 0.01% (v/v) acetone as mock control, stratified for 3 d at 4°C in darkness, treated with 3 h white light (~150 µmol $m^{-2}s^{-1}$) at 21°C, returned to darkness for 21 h at 21°C, and then grown under continuous red light (~30 µmol $m^{-2}s^{-1}$) at 21°C for 4 d before being photographed. Hypocotyl length was measured using ImageJ software (NIH).

Branching assay

The position of plants within flats was randomized to account for environmental variation. The number of primary rosette branches, not including the primary shoot, at least 1 cm in length was measured for each plant at global proliferative arrest (~7 weeks after germination).

RT-qPCR analysis

Total RNA was prepared and DNase-treated with the Spectrum Plant Total RNA Kit and On-Column DNase I Digestion Set (Sigma-Aldrich) from non-elongated axillary buds collected 10 d after anthesis or 7-d-old seedlings. First-strand cDNA was synthesized from 2 µg of total RNA with the iScript cDNA Synthesis Kit (Bio-Rad). Quantitative PCR was performed in a CFX384 Real-Time PCR Detection System (Bio-Rad) using Luna Universal qPCR Master Mix (NEB) with the following program: 5 min at 95°C and 45 cycles of 20 s at 95°C, 20 s at 60°C, and 20 s at 72°C, followed by melt curve analysis to analyze product specificity. The relative gene expression was calculated by $2^{\Delta Cq}$ [$\Delta C_q = C_q(\text{reference}) - C_q(\text{target})$]. Primers for *BRC1*, *RD29A*, *Cor15A*, *PKS5* and the *CACS* reference gene are previously described (Fujii et al. 2011; Wang et al. 2014; Soundappan et al., 2015).

Yeast Two-Hybrid Assay

To construct plasmids for yeast two-hybrid assays, the coding sequences for *D14*, *d14*^{seto}, and *d14*^{S97A} were cloned into pDONR221 Gateway entry vector by BP clonase reaction, sequence-verified, and recombined by LR clonase reaction into Gateway yeast expression vector pDest-GBKT7 to generate BD-D14, BD-d14^{seto}, and BD-d14^{S97A}, respectively. To make GAL4 DNA activation domain (AD) constructs, the coding sequences for *SMAX1*, *SMXL2*, *SMXL7*, *SMAX1*$_{D1M}$ and *SMXL7*$_{D1M}$ were cloned into pDONR221, sequence-verified, and moved into Gateway yeast expression vector pDest-GADT7. Direct interaction of two proteins was investigated by co-transformation of the respective plasmids in the yeast strain Y2HGold (Clontech) by the standard

lithium acetate-mediated method (Gietz and Woods, 2002). The transformed yeast strains were plated on SD/-Leu-Trp medium (Clontech) at 30°C for 3 d. Interactions in yeast were tested on SD/-Leu-Trp-His and SD/-Leu-Trp-His-Ade (Clontech) medium supplemented with 2 µM $GR24^{5DS}$ or 0.02% (v/v) acetone.

Transient Expression in *Nicotiana benthamiana*

N. benthamiana plants (3 weeks old) were used to express the various construct combinations by Agrobacterium (GV3101 pMP90)–mediated transient transformation of lower epidermal leaf cells as described previously (Khosla et al., 2020a).

FRET-ABP assay

N. benthamiana leaves were sprayed with 10 µM estradiol 24 hours after infiltration of Agrobacterium to induce protein expression from *LexA*:*SMXL7-mCherry* (pAB118), *LexA*:*SMAX1-mCherry* (pAB118), *LexA*:*SMXL7-mCherry-GFP* (pAB117) or *LexA*:*SMAX1-mCherry-GFP* (pAB117) (Bleckmann et al., 2010). *D14* and *$d14^{seto}$* were cloned into binary vector pGWB505 and expressed under control of the 35S promoter in *N. benthamiana* leaves. The assay was performed 24 h after induction on a Leica TCS SP5 laser scanning confocal microscope with a 63 x/ 1.2 NA water immersion objective. The FRET-APB wizard of LAS-AF was used with the following parameters: acquisition speed 700 Hz; pinhole 60.7 µm; image format 512 x 512 pixels; zoom 6X. Regions Of Interest (ROIs) of 6 x 3.5 µm were photobleached with 10 repeated exposures (laser 561 nm, 100% power level). Images were processed using Leica the Application Suite Advanced Fluorescence software (LAS-AF). FRET Efficiency (E_{FRET}%) was measured as the increase of donor fluorescence (GFP) intensity after photobleaching of the acceptor (mCherry). $E_{FRET}\% = 100 * (D_{post} - D_{pre})/ D_{post}$. D_{pre} and D_{post} were the fluorescence intensity of the donor before and after photobleaching, respectively, which were quantified using a secondary ROI inside the bleached region of the bleached region of the first ROI.

Degradation assays in *N. benthamiana*

To generate ratiometric reporter constructs for degradation assays in *N. benthamiana*, *D14*, $d14^{seto}$, and $d14^{S97A}$, *SMAX1, SMXL2*, and $SMAX1_{D2}$ entry clones were transferred into the pRATIO1212 destination vector by Gateway LR reaction (Khosla et al., 2020a). To examine the time course of degradation, the wells of a black 96-well polystyrene plate (Corning Costar®) were filled with 200 µl chemical treatments [10 µM KAR_1, 10 µM $GR24^{5DS}$, 10 µM $GR24^{ent\text{-}5DS}$, or 0.02% (v/v) acetone]. Leaf discs were excised 3 d post-infiltration and transferred to the treatment plate (one leaf disc per well) with the abaxial side up. Relative fluorescence was measured in a CLARIOstar plate reader (BMG Labtech) in plate mode (slow kinetics) at the indicated time points with the following settings: spiral scan option; scan diameter (mm), 5; and number of flashes per well per cycle, 36. Optimal settings for fluorescence measurements of the mScarlet-I reporter (ex. 560-10 nm, em. 595-10 nm) and Venus reference (ex. 497-15 nm, em. 540-20 nm) proteins were described previously (Khosla et al., 2020a). Degradation was quantified as mScarlet-I/Venus fluorescence intensity ratios after subtracting background fluorescence signals measured in leaf discs transformed with RNA silencing suppressor P19.

Degradation assays in *Arabidopsis thaliana*

4-day-old *35S*:*D14-GFP and 35S*:$d14^{seto}$*-GFP* transgenic plants (Col-0 background) grown vertically were placed in a multiwell slide (µ-Slide, 8 wells IbiTreat, IBIDI) and immobilized with 200 µl of MS agar medium [0.5% (w/v) low melting point agar]. The *rac*-GR24 was supplemented to a final concentration of 5 µM. Equivalent volume of acetone was added for mock control. 3 Z-series section images were captured every 10 min for 16 h at 22 ºC with a Microfluor Leica DMI6000B fluorescence microscope using a 10x objective and 470 nm light to detect GFP. GFP signal was quantified with Fiji using Region of Interest (ROI) Multi Measure plugging after determining a threshold range to eliminate the background. The GFP signal variation of each plant over time was calculated as a percentage to the signal at t_0 when SL was just applied. Then we

obtained the relative GFP signal by normalizing the GFP signal to the mean of its mock control at each time point.

To monitor $SMAX1_{D2}$, SMXL7 or D14 degradation, 9-day-old plants expressing *UBQ*:*SMAX1*$_{D2}$*-LUC* (pRATIO2251) (Khosla et al., 2020b), *UBQ*:*SMXL7-LUC* (pB7M34GW) or *UBQ*:*D14-LUC* (pB7M34GW) grown on a white 96-well plate (Perkin Elmer OptiPlate 96) containing 200 µl 0.5x MS agar medium were sprayed with 2 mM D-luciferin and incubated 3 h before treatment to equilibrate. 5 µM $GR24^{5DS}$, 5 µM KAR_2, 300 mM mannitol, or corresponding solvent control were then sprayed along with 2 mM D-luciferin. Luciferase signal was measured using a CLARIOstar plate reader (BMG Labtech) under controlled 21ºC temperature. The luciferase activity (LA) of each plant at each time point was calculated relative to time zero as LA (%) = $[(cps_{tn} * 100) / cps_{t0}]$. Then we obtained the Relative Luciferase Activity (RLA) by normalizing LA of each treatment/genotype to the average of corresponding solvent control at each time point.

Osmotic stress tolerance assays

7-day-old seedlings grown on 0.5x MS agar were transplanted to 0.5x MS agar containing 300 mM mannitol to induce osmotic stress. Seedlings were photographed and assayed after 14 d mannitol treatment. The aerial parts of 3-5 seedlings were excised and weighed. Tissues were ground by a bead mill and homogenized in extract solution (95% ethanol + 5% water) at 4 °C overnight until bleached. The total chlorophyll content was quantified by measuring the absorbance of the supernatant at 647 and 665 nm and using the formula total Chl = $(17.90 * A_{647} + 8.08 * A_{665})$/mg fresh weight (Chen et al., 2013).

Statistical analysis

Data were analyzed by using JMP Pro 13 and Excel. For multiple comparisons of means, one-way ANOVA was performed followed by Tukey's HSD test ($p < 0.05$) or Student's t-test ($p < 0.05$). Two-sided Student's t-test was conducted for comparisons of

means between two groups. Graphs were produced using Prism v7 (GraphPad Software). Box plots show the median, 25th percentile, and 75th percentile. Whiskers indicate the minimum and maximum of the data range, and individual data points are overlaid. For sample sizes with $n \leq 4$, individual data points and the mean value are shown.

SI References

Bleckmann, A., Weidtkamp-Peters, S., Seidel, C. A. M., and Simon, R. (2010). Stem cell signaling in Arabidopsis requires CRN to localize CLV2 to the plasma membrane. *Plant Physiol.* **152**:166–176.

Chen, L.-J., Wuriyanghan, H., Zhang, Y.-Q., Duan, K.-X., Chen, H.-W., Li, Q.-T., Lu, X., He, S.-J., Ma, B., Zhang, W.-K., et al. (2013). An S-domain receptor-like kinase, OsSIK2, confers abiotic stress tolerance and delays dark-induced leaf senescence in rice. *Plant Physiol.* **163**:1752–1765.

Clough, S. J., and Bent, A. F. (1998). Floral dip: a simplified method for Agrobacterium-mediated transformation of Arabidopsis thaliana. *Plant J.* **16**:735–743.

Fujii, H., Verslues, P. E., and Zhu, J.-K. (2011). Arabidopsis decuple mutant reveals the importance of SnRK2 kinases in osmotic stress responses in vivo. *Proc. Natl. Acad. Sci. U. S. A.* **108**:1717–1722.

Khosla, A., Rodriguez-Furlan, C., Kapoor, S., Van Norman, J. M., and Nelson, D. C. (2020a). A series of dual-reporter vectors for ratiometric analysis of protein abundance in plants. *Plant Direct* **4**.

Khosla, A., Morffy, N., Li, Q., Faure, L., Chang, S. H., Yao, J., Zheng, J., Cai, M. L., Stanga, J., Flematti, G. R., et al. (2020b). Structure–Function Analysis of SMAX1 Reveals Domains That Mediate Its Karrikin-Induced Proteolysis and Interaction with the Receptor KAI2. Plant Cell 32:2639–2659.

Scaffidi, A., Waters, M. T., Sun, Y. K., Skelton, B. W., Dixon, K. W., Ghisalberti, E. L., Flematti, G. R., and Smith, S. M. (2014). Strigolactone Hormones and Their Stereoisomers Signal through Two Related Receptor Proteins to Induce Different Physiological Responses in Arabidopsis. *Plant Physiol.* **165**:1221–1232.

Soundappan, I., Bennett, T., Morffy, N., Liang, Y., Stanga, J. P., Abbas, A., Leyser, O., and Nelson, D. C. (2015). SMAX1-LIKE/D53 Family Members Enable Distinct MAX2-Dependent Responses to Strigolactones and Karrikins in Arabidopsis. *Plant Cell* **27**:3143–3159.

Wang, X.-H., Li, Q.-T., Chen, H.-W., Zhang, W.-K., Ma, B., Chen, S.-Y., and Zhang, J.-S. (2014). Trihelix transcription factor GT-4 mediates salt tolerance via interaction with TEM2 in Arabidopsis. *BMC Plant Biol.* **14**:339.

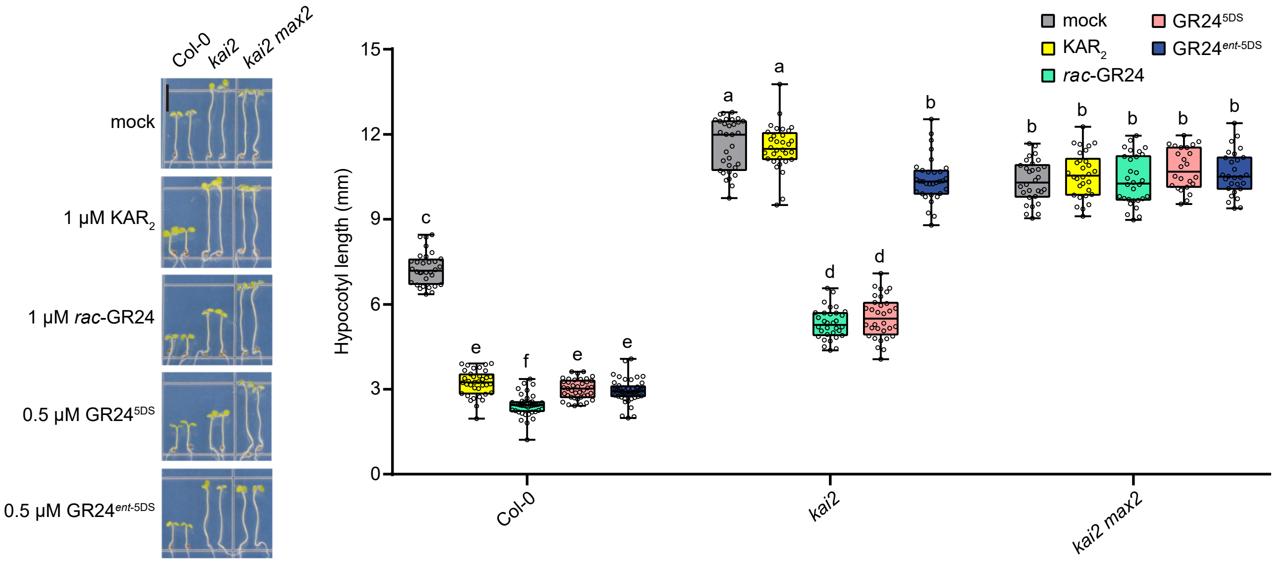

Supplemental Figure 1. Hypocotyl elongation of *kai2* is inhibited by *rac*-GR24 and GR24^{5DS} through *MAX2*.
Hypocotyl lengths of 5-d-old seedlings of Col-0 (wild type), *kai2*, and *kai2 max2* are grown under continuous red light for 4 d on the 0.5x MS agar media containing 1 μM KAR_2, 1 μM *rac*-GR24, 0.5 μM GR24^{5DS}, 0.5 μM GR24$^{ent\text{-}5DS}$ or acetone. Bar = 5 mm. Box-and-whisker plots with the same letter are not significantly different from one another (Tukey HSD, $p < 0.05$, $n \geq 30$).

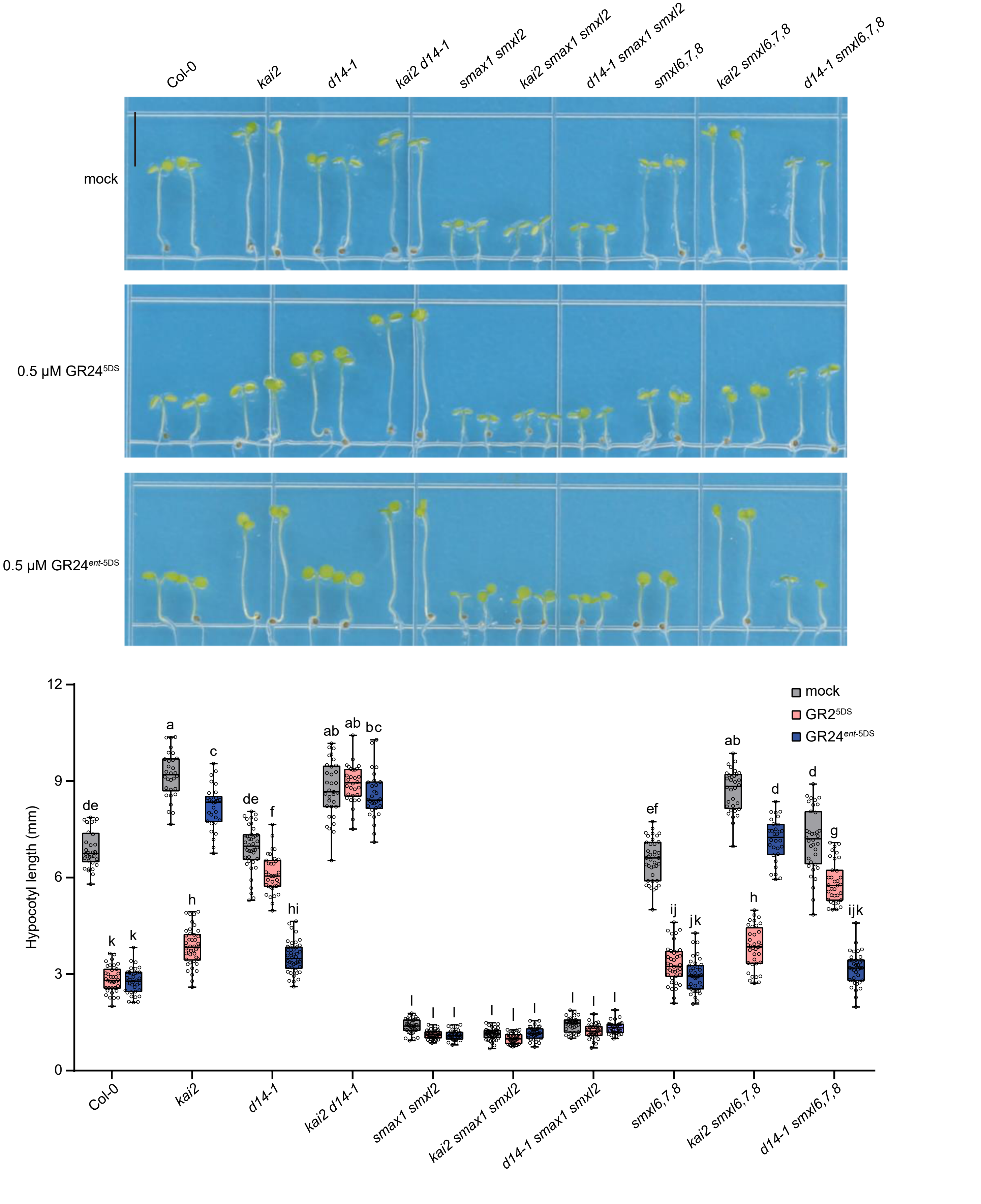

Supplemental Figure 2. Hypocotyl growth of plant materials in Figure 1B with GR24^{5DS} or GR24$^{ent\text{-}5DS}$ treatment.
Hypocotyl lengths of 5-d-old seedlings of Col-0, *kai2*, *d14-1*, *kai2 d14-1*, *smax1 smxl2*, *kai2 smax1 smxl2*, *d14-1 smax1 smxl2*, *smxl6,7,8*, *kai2 smxl6,7,8* and *d14-1 smxl6,7,8* are grown under continuous red light for 4 d on the 0.5x MS agar media containing 0.5 µM GR24^{5DS}, 0.5 µM GR24$^{ent\text{-}5DS}$ or acetone. Mock-treated seedling data are duplicated in Figure 1, which shows additional data from this experiment. Bar = 5 mm. Box-and-whisker plots with the same letter are not significantly different from one another (Tukey HSD, $p < 0.05$, $n \geq 30$).

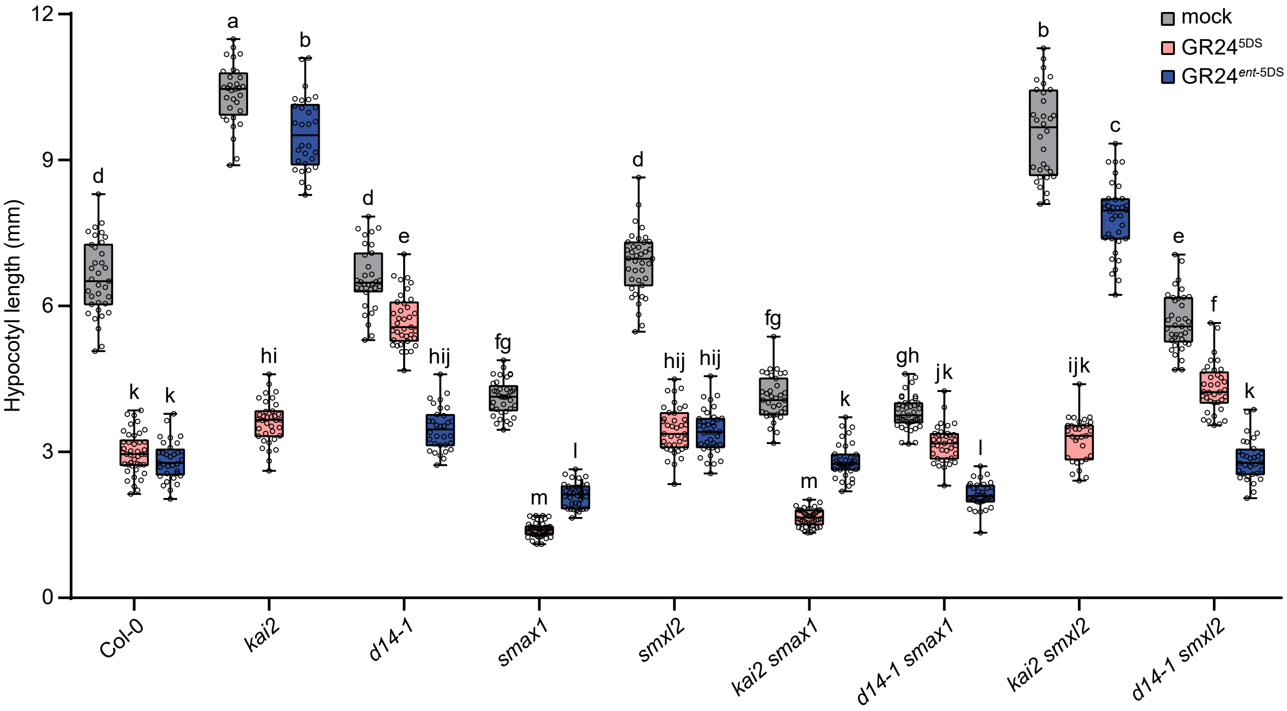

Supplemental Figure 3. Hypocotyl growth of plant materials in Figure 1C with $GR24^{5DS}$ or $GR24^{ent\text{-}5DS}$ treatment. Hypocotyl lengths of 5-d-old seedlings of Col-0, *kai2*, *d14-1, smax1, smxl2, kai2 smax1, d14-1 smax1, kai2 smxl2, d14-1 smxl2* are grown under continuous red light for 4 d on the 0.5x MS agar media containing 0.5 µM $GR24^{5DS}$, 0.5 µM $GR24^{ent\text{-}5DS}$ or acetone. Mock-treated seedling data are duplicated in Figure 1C, which shows additional data from this experiment. Box-and-whisker plots with the same letter are not significantly different from one another (Tukey HSD, $p < 0.05$, $n \geq 30$).

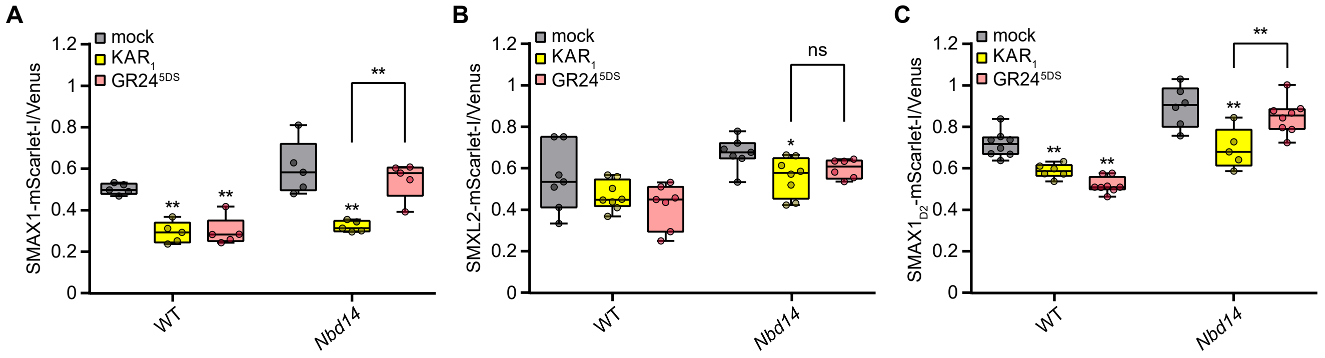

Supplemental Figure 4. Degradation of SMAX1, SMXL2 or $SMAX1_{D2}$ after 4 h treatment of KAR_1 or $GR24^{5DS}$. Relative fluorescence from the SMAX1-mScarlet-I reporter **(A)** or SMXL2-mScarlet-I reporter **(B)** or $SMAX1_{D2}$-mScarlet-I reporter **(C)** and the Venus reference after transient expression of the ratiometric system in wt tobacco and *Nbd14* is shown. Leaf discs are treated with acetone, 10 μM KAR_1, or 10 μM $GR24^{5DS}$ for 4 h. Mock-treated seedling data are duplicated in Figure 2A to 2C, which show additional data from this experiment. n = 5-8 leaf discs. ns indicates no significance. *$p < 0.05$, **$p < 0.01$, Student's t-test comparisons to the relative fluorescence at 0 h or between compared pairs.

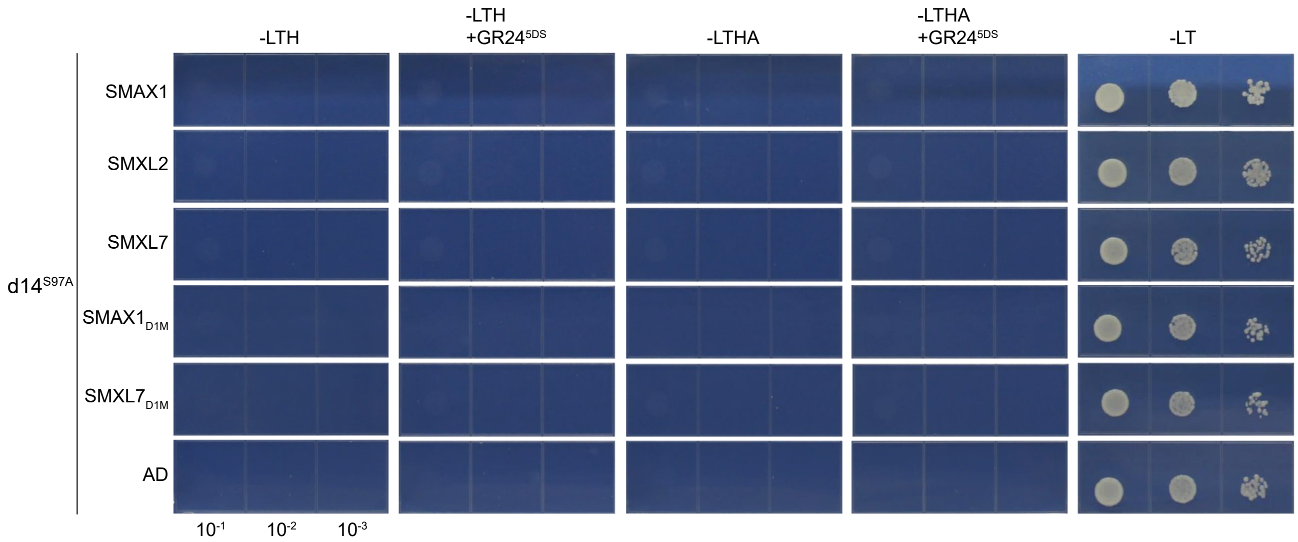

Supplemental Figure 5. Yeast two-hybrid assays for d14^{S97A} interactions with SMAX1, SMXL2, SMXL7, SMAX1$_{D1M}$ and SMXL7$_{D1M}$.

The d14^{S97A} is fused to GAL4-BD. SMAX1, SMXL7 and their D1M domains are fused to GAL4-AD. Serial 10-fold dilutions of yeast cultures are spotted onto selective growth medium (-L, -Leu; -T, -Trp; -H, -His; -A, -Ade) that is supplemented with 2 μM GR24^{5DS} or acetone.

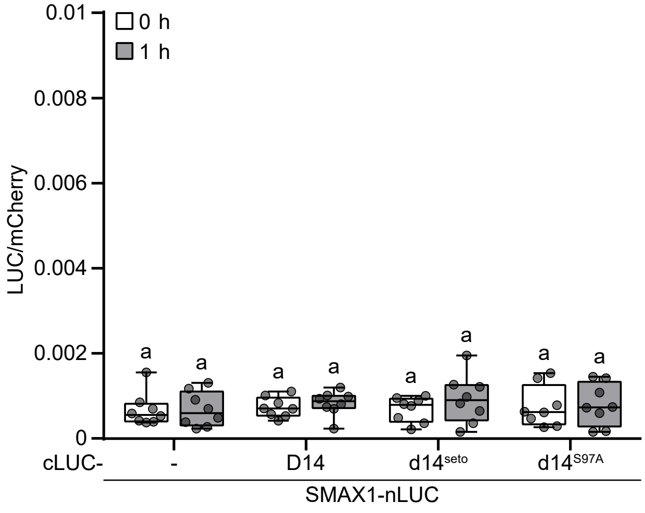

Supplemental Figure 6. D14, d14^{seto} and d14^{S97A} interactions with SMAX1 in split-luciferase assay.
N. benthamiana leaves are transiently co-transformed with *Agrobacterium tumefaciens* strains carrying cLUC, nLUC, or indicated fusions as well as a strain carrying an mCherry transgene as a transformation control. Luminescence is measured before and 1 h after treatment with 10 µM GR24^{5DS}, and normalized against mCherry fluorescence. Box-and-whisker plots with the same letter are not significantly different from one another (Student's t-test, $p < 0.05$, $n = 8$ leaf discs).

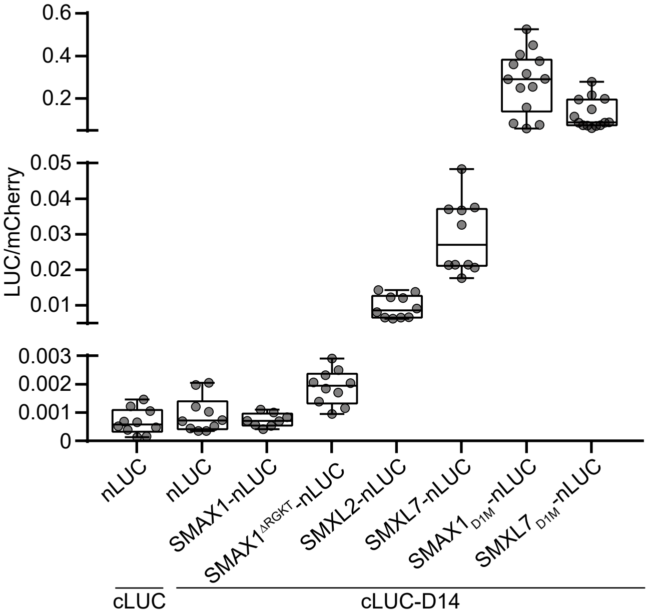

Supplemental Figure 7. Baseline of D14 interactions with SMAX1, $SMAX1^{ΔRGKT}$, SMXL2, SMXL7, $SMAX1_{D1M}$ and $SMXL7_{D1M}$ in split-luciferase assay.

N. benthamiana leaves are transiently co-transformed with *Agrobacterium tumefaciens* strains carrying cLUC, nLUC, or indicated fusions as well as a strain carrying an mCherry transgene as a transformation control. Luminescence is measured before 10 μM $GR24^{5DS}$ treatment, and normalized against mCherry fluorescence. n = 7-15 leaf discs. The data are duplicated in Figure 3B to 3F and Supplemental Figure 6, which show additional data from this experiment.

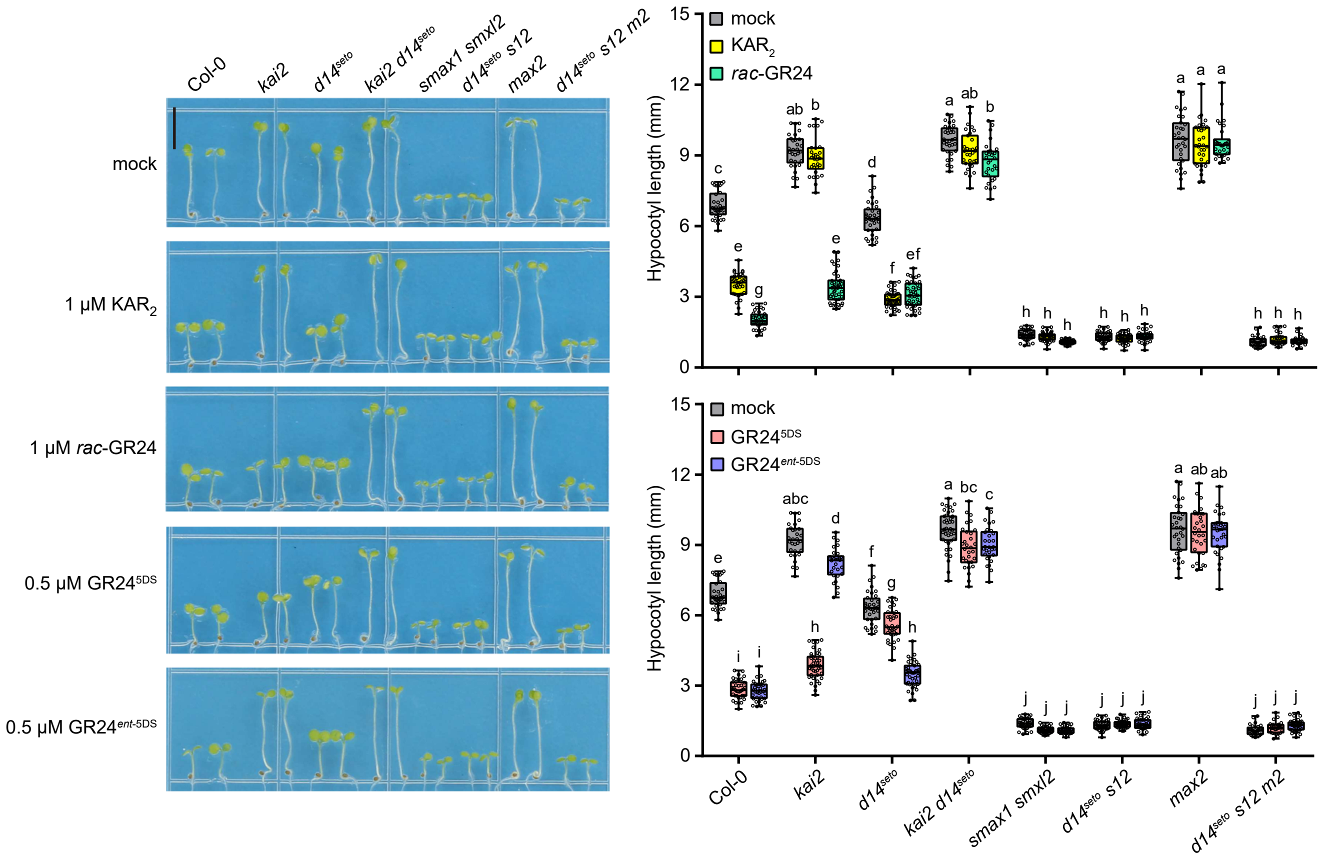

Supplemental Figure 8. Hypocotyl growth of Col-0, *kai2*, *d14^{seto}*, *kai2 d14^{seto}*, *smax1 smxl2*, *d14^{seto} smax1 smxl2*, *max2*, *d14^{seto} smax1 smxl2 max2* seedlings under different treatments.
Plants are grown under continuous red light for 4 d on the 0.5x MS agar media containing 1 μM KAR_2, 1 μM *rac*-GR24, 0.5 μM $GR24^{5DS}$, 0.5 μM $GR24^{ent\text{-}5DS}$ or acetone. Bar = 5 mm. Box-and-whisker plots with the same letter are not significantly different from one another (Tukey HSD, $p < 0.05$, $n \geq 30$).

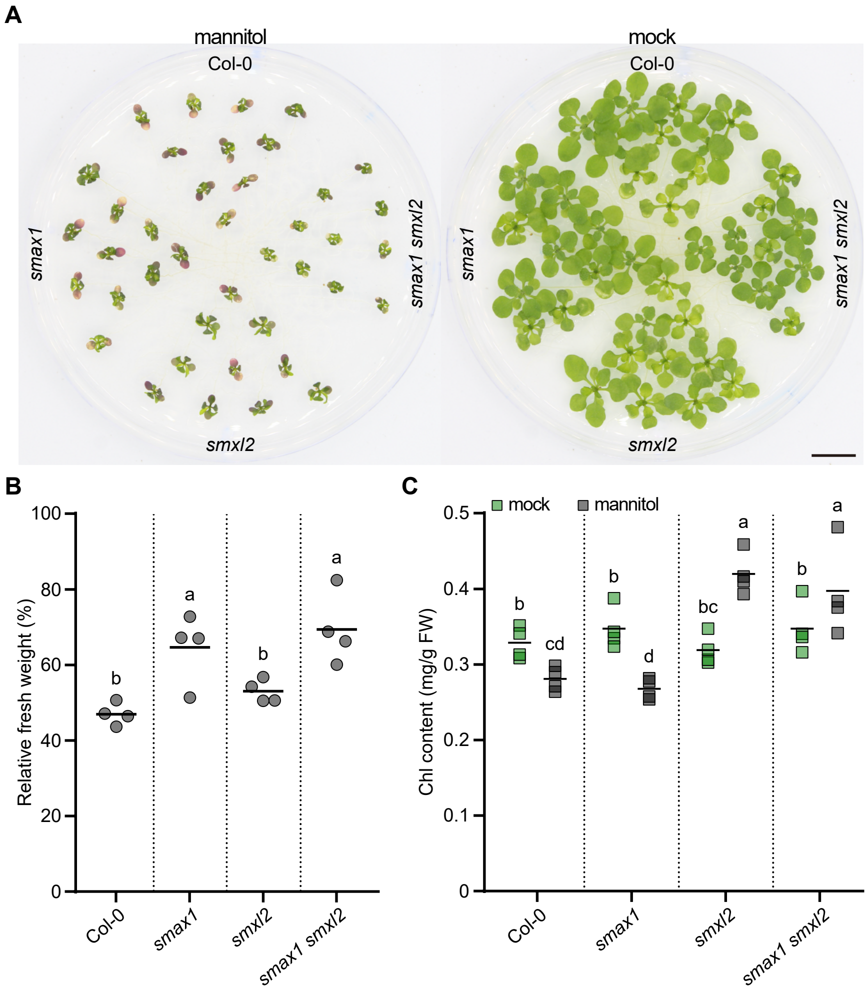

Supplemental Figure 9. Osmotic stress tolerance of Col-0, *smax1*, *smxl2* and *smax1 smxl2*.
(A) 21-day-old seedlings of Col-0, *smax1*, *smxl2* and *smax1 smxl2* grown in mock or 300 mM mannitol condition for 14 days. Bar = 1 cm.
(B) Relative fresh weights of plant materials used in **(A)** to application of 300 mM mannitol. The weights of aerial parts from plants grown on 0.5x MS agar medium containing 300 mM mannitol are scaled to that from plants grown on 0.5x MS agar medium. Scatter dot plots with the same letter are not significantly different from one another (bar indicates mean; $n = 4$; Student's t-test, $p < 0.05$).
(C) Chlorophyll (Chl) contents in the aerial parts of Arabidopsis seedlings used in **(A)**. Others are as in **(B)**.

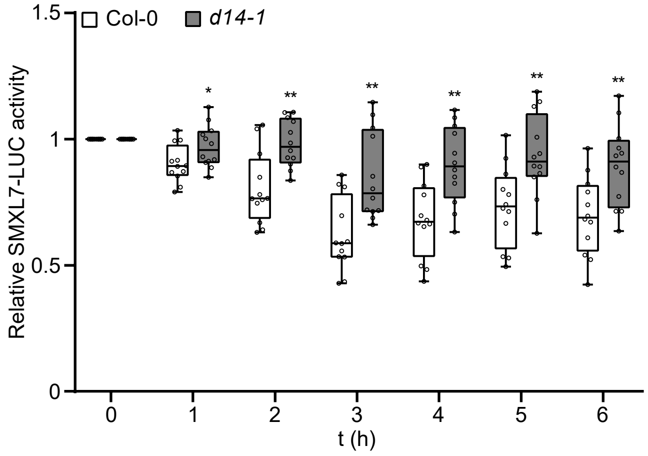

Supplemental Figure 10. Osmotic stress triggers the SMXL7 degradation.
Bioluminescence of SMXL7-LUC in Col-0 and *d14-1* backgrounds. Seedlings were treated with 300 mM mannitol or water control. Bioluminescence is shown as relative LUC activity and is monitored for 6 h after treatment. n = 12 seedlings. $*p < 0.05$, $**p < 0.01$, Student's t-test comparisons to Col-0 control at each time point.

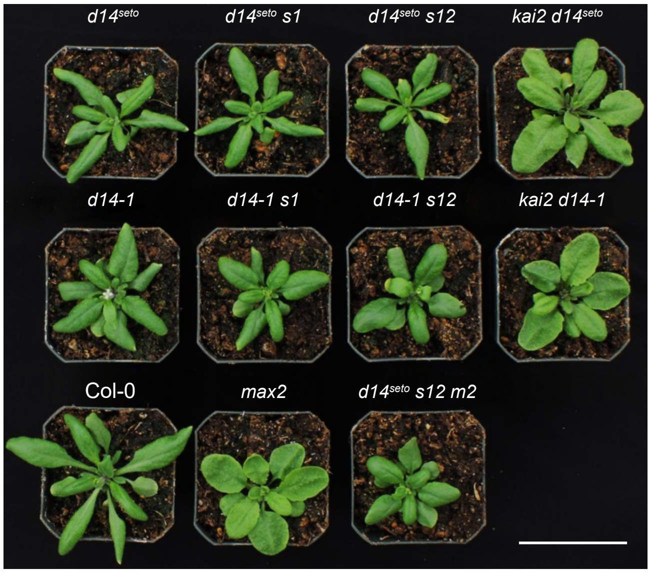

Supplemental Figure 11. Rosette phenotypes of plant materials in Figure 4A.
Col-0, *d14-1*, *kai2 d14-1*, *d14-1 smax1*, *d14-1 smax1 smxl2*, *d14*^{seto}, *kai2 d14*^{seto}, *d14*^{seto} *smax1*, *d14*^{seto} *smax1 smxl2*, *max2* and *d14*^{seto} *smax1 smxl2 max2* plants are grown for 4 weeks under a long-day photoperiod (16 h light/8 h dark) before imaging. Bar = 5 cm.

Supplemental Table 1. Primers used in this study.

Primer Name	Primers Sequences
**Primers for genotyping**	
kai2-F	CACTTGGTTCCACATCTGGTC
kai2-R	GAGATTTGAGTAACGATCGAAGTCG
max2-1_dCAPS-F	TGTCCGAATTTGGAAGAGATTAGG
max2-1_dCAPS-R	CAAGAAGAATCTTTCCCATAAACTCGAAT
d14-1-WiscLoxHS-LP	AAGAATATGGCAAGTGCAAC
d14-1-WiscLoxHS-RP	GATGATTCCGATCATAGCG
L4_WiscLoxHS	TGATCCATGTAGATTTCCCGGACATGAAG
smax1-2-Salk-LP	GTGGCAACTGTTTAGGCTGAG
smax1-2-Salk-RP	AAGCTAGCTTTTCAAGTCCCG
smxl6-4-Salk-LP	AGCCAGAGAAAGACTCGAACC
smxl6-4-Salk-RP	TCCGAAATTAAGCTCGATGTG
smxl8-1-Salk-LP	GAATCACAAATTCTGCATGGC
smxl8-1-Salk-RP	CTGACGAAGCTCCACTTTCAC
Salk-LBb1.3	ATTTTGCCGATTTCGGAAC
smxl7-3-WiscDsLox-LP	GATCAAGAAACGAACGCTGAG
smxl7-3-WiscDsLox-RP	CGTATTAGCCTCTCGGATTCC
WiscDsLox-LB-p745	AACGTCCGCAATGTGTTATTAAGTTGTC
smxl2-1-Sail-LP	TGACATACACCGATCACCAC
smxl2-1-Sail-RP	GTATCATCATCCCACTTTGCATAC
Sail-LB1	GCCTTTTCAGAAATGGATAAATAGC
seto5_dCAPS-F	GGAGGATTCGAAGAAGGTGAGATTG
seto5_dCAPS-R	CGTACGCATATTAAACAAAGTACGGC
**Primers for constructs**	
D14-cLUC-F	TCGTACGCGTCCCGGGGCATGAGTCAACACAACATCTTAGAA
D14-cLUC-R	CGAACGAAAGCTCTGCAGTCACCGAGGAAGAGCTCGCC
SMAX1-nLUC-F	AACACGGGGGACGAGCTCATGAGAGCTGGTTTAAGTACGAT
SMAX1-nLUC-R	GGACGCGTACGAGATCTGTACTGCCAAAGTAATAGTTGTCG
SMXL2-nLUC-F	GAGAGAACACGGGGGACGAGCTCATGAGAGCAGATTTGATTACTATACAGC
SMXL2-nLUC-R	CCGGGACGCGTACGAGATCTG AACGACCACCGTCCTGATACTAC
SMXL7-nLUC-F	AACACGGGGGACGAGCTCATGCCGACACCAGTAACCACG
SMXL7-nLUC-R	GGACGCGTACGAGATCTGGATCACTTCGACTCTCGCCGG
$SMAX1_{D1M}$-nLUC-F	GAGAGAACACGGGGGACGAGCTCATGTTACAACAGAACGCTTCGTC
$SMAX1_{D1M}$-nLUC-R	CCGGGACGCGTACGAGATCTGGATGTTATTATTGTTCTGCACTGATTCAG
$SMXL7_{D1M}$-nLUC-F	GAGAGAACACGGGGGACGAGCTCATGGACATTAAACTCGACGTGCTTCATC
$SMXL7_{D1M}$-nLUC-R	CCGGGACGCGTACGAGATCTGTGGTTCTTGGTTCTTCGATGCGTAG
**Primers for qRT-PCR**	
BRC1-F	TCGCGACAACCCTTTCTCACCAT
BRC1-R	CGGTCGTGTTAGTATTGCTGCCTCT
RD29A-F	GCCGACGGGATTTGACG
RD29A-R	GCCGACGGGATTTGACG
Cor15A-F	ATGGCGATGTCTTTCTCAGGAGCTGTT
Cor15A-R	TTTTATCCGTCACGAAATCTGAAGCTT

PKS5-F	GTTTGCGAGAGAGGAGAATCTG
PKS5-R	CCACAAGCAAATCATTCAACCG
CACS-F	GGAGAAGAGAGGGCCTTGCTTACAA
CACS-R	TTAGCTGGGCGAGATTTCATTTCTG
