# Supplementary material for: The strigolactone receptor D14 targets SMAX1 for degradation in response to GR24 treatment and osmotic stress
Source: Plant Commun. 2022 Jan 31;3(2):100303. doi: 10.1016/j.xplc.2022.100303 (PMC9073322; doi:10.1016/j.xplc.2022.100303)
Supplement: Document S2. Article plus supplemental information [file mmc2.pdf]

Plant Communications
Research Article

CellPress
Partner Journal

The strigolactone receptor D14 targets SMAX1 for degradation in response to GR24 treatment and osmotic stress

Qingtian Li[1], Elena Sánchez Martín-Fontecha[2], Aashima Khosla[1], Alexandra R.F. White[1], Sunhyun Chang[1], Pilar Cubas[2] and David C. Nelson[1,*]

[1]Department of Botany and Plant Sciences, University of California, Riverside, CA 92521, USA

[2]Plant Molecular Genetics Department, Centro Nacional de Biotecnología/CSIC, Campus Universidad Autónoma de Madrid, Madrid, Spain

***Correspondence: David C. Nelson (david.nelson@ucr.edu)**

https://doi.org/10.1016/j.xplc.2022.100303

ABSTRACT

The effects of the phytohormone strigolactone (SL) and smoke-derived karrikins (KARs) on plants are generally distinct, despite the fact that they are perceived through very similar mechanisms. The homologous receptors DWARF14 (D14) and KARRIKIN-INSENSITIVE2 (KAI2), together with the F-box protein MORE AXILLARY GROWTH2 (MAX2), mediate SL and KAR responses, respectively, by targeting different SMAX1-LIKE (SMXL) family proteins for degradation. These mechanisms are putatively well-insulated, with D14-MAX2 targeting SMXL6, SMXL7, and SMXL8 and KAI2-MAX2 targeting SMAX1 and SMXL2 in *Arabidopsis thaliana*. Recent evidence challenges this model. We investigated whether D14 can target SMAX1 and whether this occurs naturally. Genetic analysis indicates that the SL analog GR24 promotes D14-SMAX1 crosstalk. Although D14 shows weaker interactions with SMAX1 than with SMXL2 or SMXL7, D14 mediates GR24-induced degradation of SMAX1 in plants. Osmotic stress triggers SMAX1 degradation, which is protective, through SL biosynthesis and signaling genes. Thus, D14-SMAX1 crosstalk may be beneficial and not simply a vestige of the evolution of the SL pathway.

Key words: phytohormone, signaling, proteolysis, crosstalk

Li Q., Martín-Fontecha E.S., Khosla A., White A.R.F., Chang S., Cubas P., and Nelson D.C. (2022). The strigolactone receptor D14 targets SMAX1 for degradation in response to GR24 treatment and osmotic stress. Plant Comm. **3**, 100303.

INTRODUCTION

Strigolactones (SLs) and karrikins (KARs) are two classes of butenolide molecules that regulate diverse aspects of plant development. SLs were discovered in root exudates as germination stimulants of root-parasitic plants (Cook et al., 1966; Bouwmeester et al., 2021). SLs exuded into soil promote symbiotic interactions between roots and arbuscular mycorrhizal fungi, partly by stimulating hyphal branching (Akiyama et al., 2005; Gomez-Roldan et al., 2008; Kobae et al., 2018). SLs are also plant hormones with many roles, including the regulation of shoot branching, root growth, cambial growth, senescence, defense, and anthocyanin biosynthesis (Gomez-Roldan et al., 2008; Umehara et al., 2008; Agusti et al., 2011; Rasmussen et al., 2012; Van Ha et al., 2014; Yamada et al., 2014; Soundappan et al., 2015; Ueda and Kusaba, 2015; Lahari et al., 2019; Nasir et al., 2019; Villaécija-Aguilar et al., 2019; Wang et al., 2020a; Li et al., 2020b; Kalliola et al., 2020). KARs are abiotic signals found in smoke and biochar (Flematti et al., 2004; Kochanek et al., 2016). They promote germination of many plant species after fire but can also stimulate species from non-fire-prone environments such as *Arabidopsis thaliana* (Flematti et al., 2004; Nelson et al., 2012). In addition, KAR signaling influences seedling photomorphogenesis, mesocotyl elongation, root and root hair growth, and abiotic stress responses (Jain et al., 2006; Nelson et al., 2010; Li et al., 2017, 2020b; Wang et al., 2018; Swarbreck et al., 2019; Villaécija-Aguilar et al., 2019; Zheng et al., 2020).

Despite their different sources and effects, SLs and KARs are perceived similarly (Blázquez et al., 2020). The core SL signaling pathway in angiosperms consists of the receptor DWARF14 (D14)/DECREASED APICAL DOMINANCE2 (DAD2)/RAMOSUS3 (RMS3), the F-box protein DWARF3 (D3)/MORE AXILLARY GROWTH2 (MAX2), and transcriptional co-repressors in the

Published by the Plant Communications Shanghai Editorial Office in association with Cell Press, an imprint of Elsevier Inc., on behalf of CSPB and CEMPS, CAS.

This is an open access article under the CC BY license (http://creativecommons.org/licenses/by/4.0/).

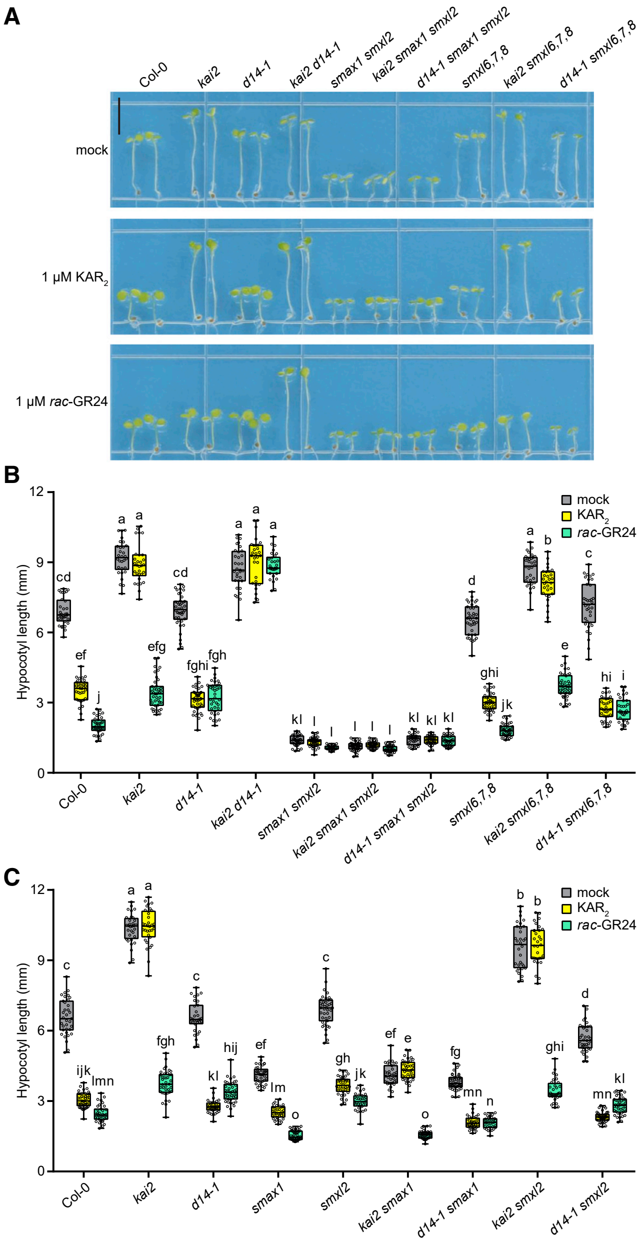

Figure 1. D14 inhibits hypocotyl growth after GR24 treatment via *SMAX1* and *SMXL2*.

(A) Images of representative 5-day-old seedlings of Col-0 (wild type), *kai2*, *d14-1*, *kai2 d14-1*, *smax1 smxl2*, *kai2 smax1 smxl2*, *d14-1 smax1 smxl2*, *smxl 6*,*7*,*8*, *kai2 smxl6*,*7*,*8*, and *d14-1 smxl6*,*7*,*8* grown under continuous red light for 4 days on 0.5× MS agar medium containing 1 μM KAR_2, 1 μM *rac*-GR24, or acetone. Bar, 5 mm.

(B) Hypocotyl lengths of the seedlings shown in (A).

(C) Hypocotyl lengths of 5-day-old seedlings of Col-0, *kai2, d14-1*, *smax1*, *smxl2*, *kai2 smax1*, *d14-1 smax1*, *kai2 smxl2*, and *d14-1 smxl2* grown under continuous red light for 4 days on 0.5× MS medium containing 1 μM KAR_2, 1 μM *rac*-GR24, or acetone. Box-and-whisker plots with the same letter are not significantly different from one another (Tukey's honest significant difference [HSD], $p < 0.05$, $n \geq 30$).

SUPPRESSOR OF MAX2 1 (SMAX1)-LIKE (SMXL) family that are known as DWARF53 (D53) in rice (*Oryza sativa*) or SMXL6, SMXL7, and SMXL8 in *Arabidopsis thaliana* (Gomez-Roldan et al., 2008; Umehara et al., 2008; Hamiaux et al., 2012; Waters et al., 2012; Jiang et al., 2013; Stanga et al., 2013; Zhou et al., 2013; de Saint Germain et al., 2016). D14 is an α/β-hydrolase that cleaves an enol-ether-linked methylbutenolide "D-ring" from SLs (Hamiaux et al., 2012; Seto et al., 2019). The D-ring becomes covalently attached to a His residue in the catalytic triad (de Saint Germain et al., 2016; Yao et al., 2016). D14 changes conformation during SL binding or hydrolysis, promoting interactions with D3/MAX2 and D53/SMXL6/7/8 (Jiang et al., 2013; Zhou et al., 2013; Wang et al., 2015; Yao et al., 2016). D14 is central to the formation of the tripartite complex, but D3 and D53 help stabilize the complex (Liang et al., 2016; Shabek et al., 2018). D3/MAX2 functions within an Skp1, Cullin, F-box (SCF)-type E3 ubiquitin ligase complex. SCF^{MAX2} polyubiquitinates D53/SMXL6/7/8 proteins, which are then rapidly degraded by the 26S proteasome (Jiang et al., 2013; Zhou et al., 2013; Soundappan et al., 2015; Wang et al., 2015; Yao et al., 2016; Shabek et al., 2018). D14 is also degraded after SL activation in a MAX2-dependent manner, but this occurs over hours rather than minutes (Chevalier et al., 2014; Hu et al., 2017).

KAR signaling shares a requirement for MAX2, but the ancient D14 paralog KARRIKIN INSENSITIVE 2 (KAI2)/HYPOSENSITIVE TO LIGHT (HTL) acts as a receptor, and SMAX1 and SMXL2 are downstream targets (Nelson et al., 2011; Sun and Ni, 2011; Waters et al., 2012; Stanga et al., 2013, 2016; Khosla et al., 2020b; Zheng et al., 2020). Similar to SL signaling, the activation of KAI2 triggers its association with MAX2 and SMAX1/SMXL2, leading to SMAX1 and SMXL2 degradation (Yao et al., 2017; Xu et al., 2018; Carbonnel et al., 2020a; Khosla et al., 2020b; Wang et al., 2020b, 2021; Zheng et al., 2020). Polyubiquitination has been demonstrated for SMXL2 and is presumed for SMAX1 (Wang et al., 2020b). KAI2 is also degraded after activation, although unlike D14, this is SMAX1/SMXL2- dependent rather than MAX2-dependent (Waters et al., 2015b; Khosla et al., 2020b). In addition to mediating KAR responses, KAI2 is thought to recognize an endogenous signal, KAI2 ligand (KL), that remains undiscovered (Waters et al., 2015a; Conn and Nelson, 2015). KAI2 is more sensitive to desmethyl butenolide compounds than methylbutenolide compounds, which may give hints about the chemical structure of KL (Yao et al., 2021). KARs themselves are likely to require metabolism in plants for recognition by KAI2 (Waters et al., 2015a; Xu et al., 2018; Khosla et al., 2020b; Wang et al., 2020b; Nelson, 2021).

There is substantial evidence that SL and KAR/KL pathways function independently despite their homology. First, SL and KAR treatments usually affect different aspects of plant growth (Waters et al., 2017). For example, SLs inhibit shoot branching, whereas KARs promote Arabidopsis germination (Nelson et al., 2011; Scaffidi et al., 2014). Second, genetic analysis often shows different roles for SL and KAR/KL pathway genes. SL-insensitive and SL-deficient mutants often have different phenotypes than the KAR/KL-insensitive mutant *kai2* (Nelson et al., 2011; Waters et al., 2012; Villaécija-Aguilar et al., 2019). Likewise, *smax1* (or *smax1 smxl2*) and *smxl6,7,8* mutants suppress different *max2* phenotypes that are associated with KAR/KL and SL insensitivity, respectively (Stanga et al., 2013, 2016; Soundappan et al., 2015; Wang et al., 2015; Swarbreck

et al., 2019; Villaécija-Aguilar et al., 2019). In some cases, however, such as drought resistance or mesocotyl elongation, both pathways may influence a trait (Li et al., 2020b; Zheng et al., 2020). Third, promoter-swapping experiments show that *KAI2* and *D14* are not interchangeable genes whose unique roles arise from different expression patterns (Waters et al., 2015a; Carbonnel et al., 2020b). Fourth, D14 and KAI2 prefer to interact with different SMXL targets (Yao et al., 2017; Khosla et al., 2020b; Wang et al., 2020b; Zheng et al., 2020). Receptor-SMXL interaction specificity is linked to the central D1M domains of SMXL proteins (Khosla et al., 2020b). Fifth, KAR treatment triggers degradation of SMAX1-type, but not D53-type, SMXL proteins (Jiang et al., 2013; Wang et al., 2015; Khosla et al., 2020b; Zheng et al., 2020). Transient co-expression of SL and KAR/KL signaling components from *Lotus japonicus* in *Nicotiana benthamiana* also suggests the specific degradation of SMAX1 by KAI2 and a D53-type SMXL by D14 (Carbonnel et al., 2020a). Finally, evolutionary analysis indicates that D14 was derived from KAI2 and D53-type SMXL proteins were derived from SMAX1-type SMXLs (Bythell-Douglas et al., 2017; Walker et al., 2019). Co-evolution of D14 and D53-type SMXLs may have produced an orthogonal SL signaling pathway.

Recent work has challenged the model of insulated SL and KAR pathways. Genetic studies of lateral root development and root skewing initially implied that KAI2 may target SMXL6, SMXL7, and SMXL8 (Swarbreck et al., 2019). However, lateral root development was later shown to be regulated additively by SL and KAR/KL pathways, putatively with shifting contributions from each at different developmental stages (Villaécija-Aguilar et al., 2019). The effect of *smxl6,7,8* on root skewing, which is KAI2-regulated, has been inconsistent between different labs (Swarbreck et al., 2019; Villaécija-Aguilar et al., 2019). Thus, there is no strong support for KAI2-SMXL6,7,8 crosstalk. By contrast, there is compelling biochemical evidence that D14 can target SMXL2 (Wang et al., 2020b). SMXL2 co-immunoprecipitates D14 in the presence of $GR24^{5DS}$ or $GR24^{4DO}$, synthetic SL analogs of the natural SLs 5-deoxystrigol (5DS) and 4-deoxyorobanchol (4DO). Furthermore, $GR24^{4DO}$ promotes the polyubiquitination and degradation of SMXL2 through D14 in the *kai2* background (Wang et al., 2020b). This indicates that one-way crosstalk between the SL and KAR pathways is possible, while also raising the question of whether it occurs naturally.

Co-immunoprecipitation of D14 by SMAX1 was not observed, and it is unknown whether D14 can stimulate SMAX1 degradation (Wang et al., 2020b). However, the potential for D14-SMAX1 crosstalk has been suggested by D14-mediated effects of GR24 on hypocotyl elongation, root-hair density, and root-hair elongation, which are controlled by SMAX1 and SMXL2 (Waters et al., 2012; Toh et al., 2014; Stanga et al., 2016; Villaécija-Aguilar et al., 2019). We investigated whether D14 can interact with SMAX1 and target it for degradation. Here, we report that *KAI2*-independent hypocotyl inhibition in the presence of an SL analog is genetically dependent on *D14* and *MAX2* and is primarily due to the destabilization of SMAX1. Although the ability of D14 to interact with SMAX1 and SMXL2 may be a little-used vestige of its evolution from KAI2, this crosstalk has physiological relevance for osmotic stress responses in seedlings.

RESULTS

Genetic evidence for D14 crosstalk with SMAX1 and SMXL2 in seedlings

KAR_1, KAR_2, and *rac*-GR24 (a racemic mixture of $GR24^{5DS}$ and $GR24^{ent-5DS}$) inhibit hypocotyl elongation of Arabidopsis seedlings grown under continuous red light (Nelson et al., 2010). $GR24^{5DS}$ has a D-ring in the stereochemical configuration of natural SLs and signals through D14. Its enantiomer, $GR24^{ent-5DS}$, has a D-ring configuration that is not found in SLs. $GR24^{ent-5DS}$ signals mostly through KAI2 but can also activate D14 *in vitro* and *in vivo* (Scaffidi et al., 2014; Waters et al., 2015a; Flematti et al., 2016). Although *kai2* seedlings are insensitive to KAR_2 and mostly insensitive to $GR24^{ent-5DS}$, responses to *rac*-GR24 and $GR24^{5DS}$ remain (Waters et al., 2012; Scaffidi et al., 2014). We first tested whether KAI2-independent responses to GR24 require MAX2. *rac*-GR24 and $GR24^{5DS}$ had no effect on the *kai2 max2* hypocotyl, confirming that responses to these compounds are *MAX2*-dependent (Supplemental Figure 1).

We next examined genetic interactions among *kai2*, *d14-1*, *smax1*, and *smxl* mutants to determine which *SMXL* genes are epistatic to *KAI2* and *D14* (Figures 1A and 1B; Supplemental Figure 2). As shown previously, *d14-1* showed wild-type hypocotyl elongation under control conditions, implying that endogenous SLs do not affect hypocotyl growth. By contrast, *kai2* had elongated hypocotyls, and *smax1 smxl2* hypocotyls were very short (Waters et al., 2012; Stanga et al., 2016). The *kai2 d14-1* double mutant was similar to *kai2* but was also insensitive to GR24 treatments, indicating that *KAI2*-independent responses to GR24 occur through *D14* (Scaffidi et al., 2014). The *kai2 smax1 smxl2* and *d14-1 smax1 smxl2* triple mutants showed dramatically decreased hypocotyl lengths that were not further affected by KAR_2 or GR24 treatments, similar to *smax1 smxl2*. This indicated that *SMAX1* and *SMXL2* are epistatic to *KAI2* (Figures 1A and 1B).

Because hypocotyl elongation of *d14-1* is similar to that of the wild type, however, the *d14-1 smax1 smxl2* triple mutant did not clarify whether SMAX1 and SMXL2 also act downstream of D14 or function in a separate pathway. We found evidence for the former idea by excluding a role for *SMXL6*, *SMXL7*, and *SMXL8* in hypocotyl growth. We did not observe an appreciable difference between *smxl6,7,8* and wild-type seedlings under the mock condition or in their responses to KAR_2 or GR24 (Figures 1A and 1B). Moreover, *smxl6,7,8* mutations did not substantially affect the length of *kai2* or *d14-1* hypocotyls under the mock condition or their responses to KAR_2 and GR24 treatments, in clear contrast to *smax1 smxl2*. Therefore, D14-mediated responses to *rac*-GR24 and $GR24^{5DS}$ in seedling hypocotyls are not due to SMXL6,7,8 degradation. Instead, D14 is likely to target SMAX1 and/or SMXL2 for degradation in the presence of GR24.

SMAX1 is the primary regulator of hypocotyl growth targeted by KAI2 and D14

Given the biochemical evidence for D14 interactions with SMXL2, but not SMAX1, we hypothesized that D14 may target SMXL2 for degradation more effectively than SMAX1 (Wang

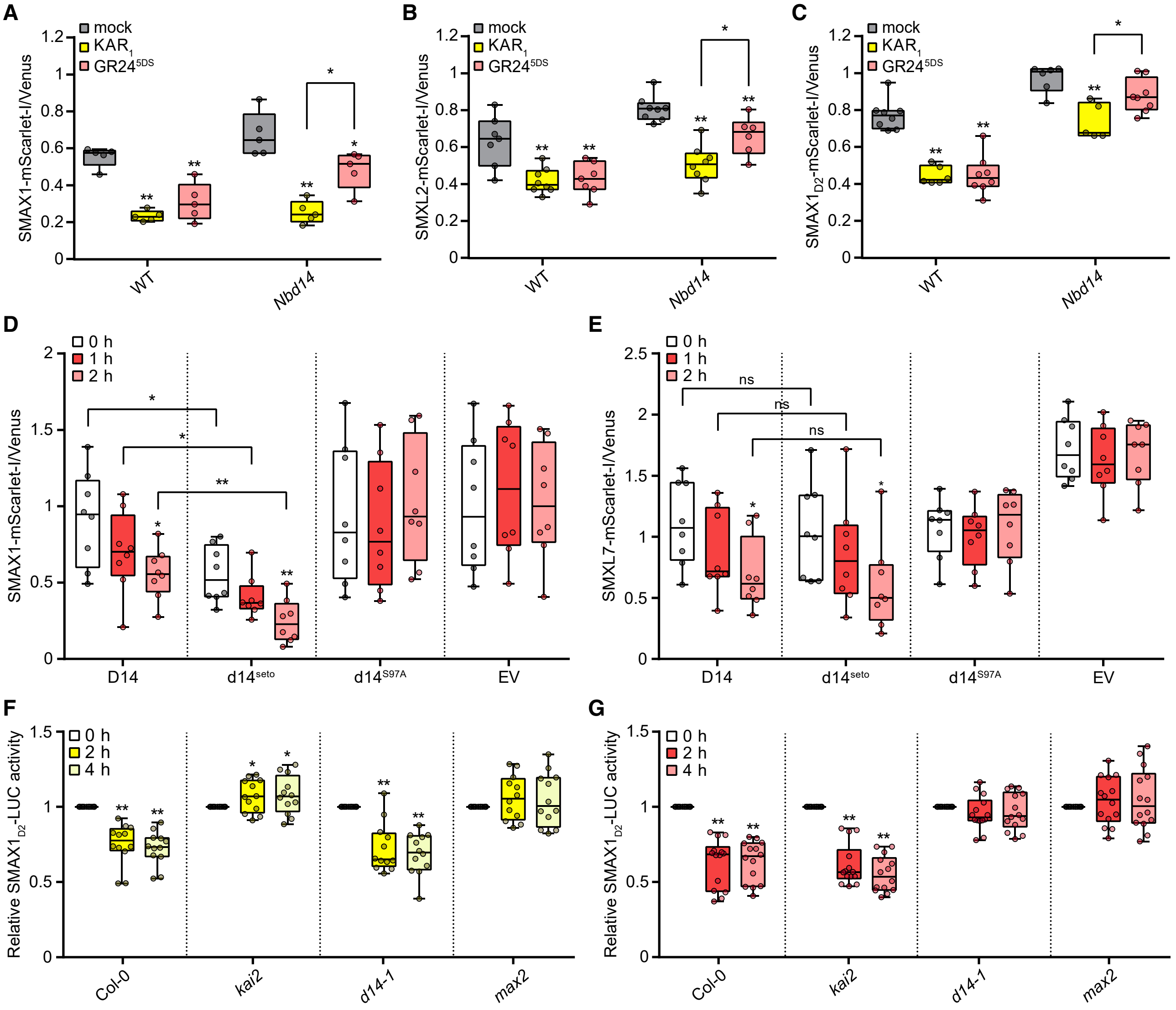

Figure 2. SL triggers SMAX1 and SMXL2 degradation through D14.

(A–C) Relative fluorescence from the SMAX1-mScarlet-I reporter **(A)**, the SMXL2-mScarlet-I reporter **(B)**, or the SMAX1$_{D2}$-mScarlet-I reporter **(C)** and the Venus reference after transient expression of the ratiometric system in wild-type (WT) tobacco and *Nbd14* is shown. Leaf discs were treated with acetone, 10 μM KAR$_1$, or 10 μM GR24^{5DS} for 12 h before measurement. n = 5–8 leaf discs. Asterisks indicate significant differences from each acetone control or between compared pairs using Student's t test (*$p < 0.05$ and **$p < 0.01$).

(D and E) Relative fluorescence from the SMAX1-mScarlet-I reporter **(D)** or the SMXL7-mScarlet-I reporter **(E)** along with D14, d14^{seto}, d14^{S97A}, or an empty vector (EV) expressed in *Nbd14* at 0, 1, and 2 h after 10 μM GR24^{5DS} treatment. n = 12 leaf discs. ns, no significance. *$p < 0.05$, **$p < 0.01$, Student's t test comparisons with the relative fluorescence at 0 h or between compared pairs.

(F and G) SMAX1$_{D2}$-luciferase (LUC) transgenic seedlings in the Col-0, *kai2*, *d14-1*, and *max2* backgrounds were treated with 5 μM KAR$_2$ **(F)**, 5 μM GR24^{5DS} **(G)**, or acetone for 4 h. Bioluminescence is shown as relative LUC activity at 0, 2, and 4 h after treatment. n = 12–14 seedlings. *$p < 0.05$, **$p < 0.01$, Student's t test comparisons with each genotype/treatment at 0 h.

et al., 2020b). To assess whether KAI2 and D14 differentially target SMAX1 and SMXL2 during hypocotyl elongation, we compared the growth of *d14-1 smax1*, *d14-1 smxl2*, *kai2 smax1*, and *kai2 smxl2* seedlings (Figure 1C). Consistent with the larger role of SMAX1 in hypocotyl elongation, *smax1* dramatically suppressed the elongated hypocotyl phenotype of *kai2*, whereas *smxl2* had little effect (Stanga et al., 2016). Responses to *rac*-GR24 and GR24^{5DS} were similarly strong in *kai2 smxl2* and *kai2*, putatively reflecting the ability of D14 to act upon SMAX1 (Figure 1C and Supplemental Figure 3). Interestingly, the average hypocotyl length of seedlings treated with *rac*-GR24 was slightly shorter for *kai2 smax1* (in which D14 and SMXL2 remain) than for *d14-1 smax1* (in which KAI2 and SMXL2 remain), suggesting that D14 may target SMXL2 better than KAI2. Conversely, the hypocotyl length of seedlings treated with *rac*-GR24 was slightly longer for *kai2 smxl2* than for *d14-1 smxl2*, suggesting that KAI2 may target SMAX1 better than D14 (Figure 1C). A similar pattern of results was observed in treatments with purified GR24 stereoisomers (Supplemental Figure 3).

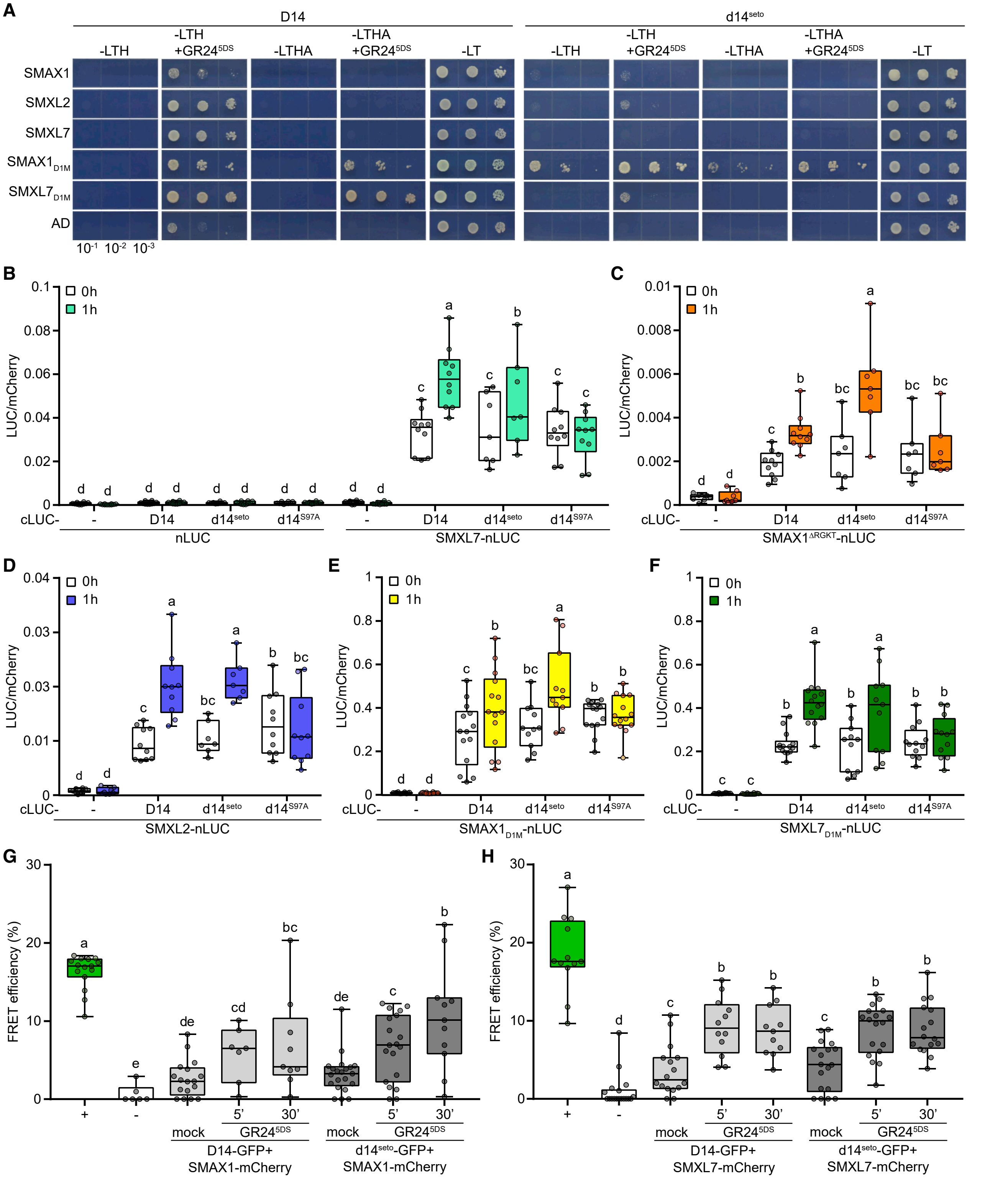

Figure 3. D14 and SMAX1 proteins can physically interact.

(A) Yeast two-hybrid assays for D14 and d14^{seto} interactions with SMAX1, SMXL7, and their D1M domains. D14 and d14^{seto} were fused to GAL4-BD. SMAX1, SMXL7, and their domains were fused to GAL4-AD. Serial 10-fold dilutions of yeast cultures were spotted onto selective growth medium (-L, -Leu; -T, -Trp; -H, -His; -A, -Ade) supplemented with 2 μM GR24^{5DS} or acetone (control).

(B–F) Split-LUC complementation assay for interactions between SMXL7 **(B)**, SMAX1$^{\Delta RGKT}$ **(C)**, SMXL2 **(D)**, and D1M domains of SMAX1 **(E)** and SMXL7 **(F)** with D14, d14^{seto}, or d14^{S97A}. *N. benthamiana* leaves were transiently co-transformed with *Agrobacterium tumefaciens* strains carrying cLUC, nLUC,

(legend continued on next page)

SMAX1 is degraded after $GR24^{5DS}$ treatment by $D14\text{-}SCF^{MAX2}$ signaling

We next used a ratiometric reporter system to investigate whether D14 can induce degradation of Arabidopsis SMAX1 and SMXL2 proteins (Figure 2) (Khosla et al., 2020a, 2020b). We transiently expressed pRATIO1212-SMAX1, -SMXL2, and -$SMAX1_{D2}$ (a C-terminal domain of SMAX1 sufficient for degradation; see below) dual-fluorescent reporter constructs in wild-type *N. benthamiana* leaves and tested the effects of 10 μM KAR_1 and $GR24^{5DS}$ treatments on excised leaf discs. The ratio of mScarlet-I/Venus fluorescence decreased for all constructs in response to both treatments, indicating the degradation of SMAX1-, SMXL2-, and $SMAX1_{D2}$-mScarlet-I fusion proteins (Figures 2A–2C). The extent of degradation induced by $GR24^{5DS}$ was similar to that induced by KAR_1. Although $GR24^{5DS}$ responses are predominantly mediated by D14 in Arabidopsis, we could not assume that $GR24^{5DS}$-induced degradation of SMAX1 and SMXL2 in *N. benthamiana* was due to D14 alone. Therefore, we also tested these constructs in an *N. benthamiana d14a d14b* double mutant (*Nbd14*) background (White et al., 2021). The SMXL7 reporter was unaffected by *rac*-GR24 in *Nbd14*, indicating that its degradation is specifically mediated by *N. benthamiana* D14 proteins and not by KAI2 (White et al., 2021). In *Nbd14* leaves, we observed 55% and 51% less degradation of SMAX1 and SMXL2 reporters, respectively, after 12 h treatment with $GR24^{5DS}$ compared with KAR_1 (Figures 2A and 2B). At an earlier 4-h time point, $GR24^{5DS}$ had very little effect on SMAX1 degradation compared with KAR_1 in the *Nbd14* mutant, but it was effective in the wild type (Supplemental Figure 4). These results indicated that *N. benthamiana* D14 proteins mediate much, although not all, of the $GR24^{5DS}$-induced degradation of SMAX1 and SMXL2.

To verify that D14 can cause SMAX1 degradation, we rescued the *Nbd14* mutant by transient expression of Arabidopsis *D14*. As a negative control, we tested the $d14^{S97A}$ mutant, which has no SL hydrolysis or signaling activity (Waters et al., 2015a; Seto et al., 2019). We also tested the *seto5/d14-2* allele of Arabidopsis *D14* (referred to here as $d14^{seto}$ to avoid confusion with the *Osd14-2* allele in rice). The $d14^{seto}$ mutant has increased axillary bud outgrowth, similar to the loss-of-function T-DNA insertion allele *d14-1* (Chevalier et al., 2014). Co-expression of D14 restored the degradation of SMAX1 and SMXL7 reporters following $GR24^{5DS}$ treatment in *Nbd14* leaves (Figures 2D and 2E). By contrast, $d14^{S97A}$ failed to restore $GR24^{5DS}$-induced degradation of SMAX1 and SMXL7. Interestingly, $d14^{seto}$ enabled $GR24^{5DS}$-induced degradation of SMAX1 and SMXL7, similar to D14. Moreover, in the absence of $GR24^{5DS}$ treatment, $d14^{seto}$ co-expression reduced the accumulation of the SMAX1 reporter relative to *D14* co-expression (Figures 2D and 2E). Therefore, the $d14^{seto}$ allele does not cause a complete loss of function and may be more effective at triggering SMAX1 degradation.

We next investigated whether SMAX1 degradation in Arabidopsis also involves D14. We have not yet been successful in detecting full-length SMAX1 in Arabidopsis (Khosla et al., 2020b). However, the C-terminal D2 domain of SMAX1 ($SMAX1_{D2}$) is more stable than SMAX1 and is necessary and sufficient for MAX2-mediated degradation if full-length SMAX1 and/or SMXL2 proteins are also present. $SMAX1_{D2}$ lacks the central D1M domains that mediate interactions between SMXL proteins and their receptor partners, KAI2 or D14, and it is therefore likely to be targeted for degradation indirectly through association with SMAX1 or SMXL2 (Khosla et al., 2020b). D14-mediated, $GR24^{5DS}$-induced degradation of the $SMAX1_{D2}$ ratiometric reporter in *N. benthamiana* was similar to that of the full-length SMAX1 and SMXL2 reporters (Figure 2C). Therefore, we crossed *kai2* and *d14-1* mutations into a stable transgenic $SMAX1_{D2}$-luciferase (LUC) reporter line in Arabidopsis to analyze KAR_2- and $GR24^{5DS}$-induced degradation responses (Khosla et al., 2020b). KAR_2 caused a significant decline in $SMAX1_{D2}$-LUC bioluminescence within 4 h in wild-type and *d14-1* seedlings but had no effect on *kai2* or *max2* seedlings (Figure 2F). By contrast, $GR24^{5DS}$ caused a decline in the abundance of $SMAX1_{D2}$-LUC reporters in wild-type and *kai2* seedlings but not in *d14-1* or *max2* seedlings (Figure 2G). This result demonstrated that $GR24^{5DS}$-induced degradation of $SMAX1_{D2}$ (and, by proxy, SMAX1 and/or SMXL2) in Arabidopsis is due to D14 and MAX2 activity.

$GR24^{5DS}$ promotes interactions of D14 with SMAX1 and SMXL2

To determine whether D14 targets SMAX1 and SMXL2 directly, we investigated interactions among these proteins. In yeast two-hybrid (Y2H) assays, $GR24^{5DS}$ stimulated protein-protein interactions between D14 and SMAX1, SMXL2, and SMXL7. Based upon the relative growth rates of yeast under low-stringency histidine dropout selection, D14-SMAX1 interactions were weaker than D14-SMXL2 and D14-SMXL7 interactions and not very different from a GAL4 activation domain (AD) negative control. In the presence of $GR24^{5DS}$, D14 had stronger interactions with the D1M domains of SMAX1 and SMXL7 ($SMAX1_{D1M}$ and $SMXL7_{D1M}$) than the full-length proteins, as indicated by yeast growth under higher-stringency histidine and adenine dropout selection. Again, D14 showed a stronger interaction with $SMXL7_{D1M}$ than with $SMAX1_{D1M}$ (Figure 3A). As a negative

or the indicated fusions, as well as a strain carrying an mCherry transgene as a transformation control. Luminescence was measured before and 1 h after treatment with 10 μM $GR24^{5DS}$ and was normalized against mCherry fluorescence. Box-and-whisker plots with the same letter are not significantly different from one another (Student's t test, $p < 0.05$, n = 7–15 leaf discs).

(G) FRET-ABP assay for interactions of SMAX1 with D14. *N. benthamiana* leaves were transiently co-transformed with *Agrobacterium tumefaciens* strains carrying SMAX1-GFP-mCherry or the indicated fusions. The FRET efficiency is shown as the percentage increase in donor fluorescence compared with that before receptor bleaching. + (dark green box) and – (white box) indicate SMAX1-GFP-mCherry as a positive control and the SMAX1-mCherry/Myc-GFP pair as a negative control, respectively. Acetone-treated leaf discs were used as mock controls. Box-and-whisker plots with the same letter are not significantly different from one another (Student's t test, $p < 0.05$, n = 6–21 leaf discs).

(H) FRET-ABP assay for interactions of SMXL7 with D14. *N. benthamiana* leaves were transiently co-transformed with *Agrobacterium tumefaciens* strains carrying SMXL7-GFP-mCherry or the indicated fusions. + (dark green box) and – (white box) indicate SMXL7-GFP-mCherry as a positive control and the SMXL7-mCherry/Myc-GFP pair as a negative control, respectively. Acetone-treated leaf discs were used as mock controls. Box-and-whisker plots with the same letter are not significantly different from one another (Student's t test, $p < 0.05$, n = 6–18 leaf discs).

control, we tested d14^{S97A} and observed no interactions (Supplemental Figure 5).

We also investigated SMAX1 and SMXL7 interactions with d14^{seto}, which has an amino acid substitution at the solvent-exposed surface of a D14 cap helix that may influence protein-protein interactions (Chevalier et al., 2014). We observed that d14^{seto} had highly reduced or abolished Y2H interactions with SMAX1, SMXL2, SMXL7, SMXL7$_{D1M}$, and the GAL4 AD itself in the presence of GR24^{5DS} compared with D14. Unexpectedly, d14^{seto} maintained the interaction with SMAX1$_{D1M}$ and, furthermore, interacted with SMAX1$_{D1M}$ in the absence of GR24^{5DS} (Figure 3A).

To validate the Y2H results in a plant system, we examined D14 interactions with SMXL proteins using split-LUC assays in *N. benthamiana* leaves. N- and C-terminal portions of firefly LUC were fused, respectively, to the C-termini of SMXL proteins and the N-termini of D14, d14^{seto}, or d14^{S97A}. To normalize transformation efficiencies across samples, the fluorescent protein mCherry was co-expressed with the split-LUC constructs. These assays were performed in *Nbd14* leaves to avoid possible interference from native NbD14 proteins. The ratio of LUC to mCherry signal produced by cLUC-D14 and SMXL7-nLUC was significantly higher than that produced with unfused cLUC or nLUC negative controls. GR24^{5DS} further increased the LUC/mCherry ratio for D14-SMXL7, consistent with enhanced protein-protein interaction. Although d14^{S97A} produced a similar interaction with SMXL7 as D14 before treatment, GR24^{5DS} had no effect (Figure 3B). In contrast to the Y2H experiments, d14^{seto} appeared to interact with SMXL7 similarly to D14, albeit with a putatively reduced response to GR24^{5DS}. We next tested D14 interactions with SMAX1 and SMXL2. We were unable to detect a LUC/mCherry signal for D14-SMAX1 above that of the negative controls, even in the presence of GR24^{5DS} (Supplemental Figure 6). This may reflect the instability of SMAX1 (Khosla et al., 2020b). Deletion of a conserved P-loop motif (RGKT) causes resistance to SCF^{MAX2}-mediated degradation in D53-type SMXL proteins as well as SMAX1 and SMXL2 (Jiang et al., 2013; Zhou et al., 2013; Soundappan et al., 2015; Wang et al., 2015, 2020b; Liang et al., 2016; Khosla et al., 2020b). Therefore, we tested interactions between SMAX1$^{\Delta RGKT}$ and D14. This enabled the detection of a GR24^{5DS}-responsive interaction with D14, although with a much lower signal than D14-SMXL7 or D14-SMXL2. SMAX1$^{\Delta RGKT}$ and SMXL2 interactions with D14, d14^{seto}, and d14^{S97A} were qualitatively similar to those observed for SMXL7, with a positive GR24^{5DS} response maintained for d14^{seto} but not for d14^{S97A} (Figures 3C and 3D). SMAX1$_{D1M}$ and SMXL7$_{D1M}$ showed a pattern of interactions with D14 and d14 mutant proteins that was similar to that of full-length SMXL proteins but produced stronger luminescence signals (Figures 3E and 3F, Supplemental Figure 7). In contrast to the Y2H experiments, we did not observe reduced interactions between SMXL7$_{D1M}$ and d14^{seto} compared with D14 in the split-LUC assays.

The differing results in Y2H and split-LUC assays led us to further examine D14 and d14^{seto} interactions with SMAX1 and SMXL7 by measuring Förster resonance energy transfer after acceptor photobleaching (FRET-APB) (Day et al., 2001). This technique determines FRET efficiency, which is a measure of protein-protein interactions, by comparing the fluorescence of the donor (e.g., GFP) before and after photobleaching of the acceptor (e.g., mCherry). We performed FRET-APB assays with D14-GFP, d14^{seto}-GFP, and SMAX1-mCherry fusion proteins co-expressed in *N. benthamiana* leaves. Photobleaching of SMAX1-mCherry caused a negligible change in fluorescence of a myc-GFP negative control, indicating an absence of FRET between these two proteins (Figure 3G). By contrast, FRET was detected between D14-GFP and SMAX1-mCherry. After 5 min of treatment with a solvent control, SMAX1-mCherry photobleaching caused a small increase in D14-GFP fluorescence. Treatment with GR24^{5DS} for 5 or 30 min increased the FRET efficiency approximately 2- to 3-fold above the solvent control. Similar results were obtained for d14^{seto}-GFP and SMAX1-mCherry. The average FRET efficiency between d14^{seto}-GFP and SMAX1-mCherry was higher than that between D14-GFP and SMAX1-mCherry after 30 min of GR24^{5DS} treatment (10.4% versus 6.9%), although this difference was not statistically significant ($p = 0.24$, Student's t test). We also examined D14-SMXL7 interactions with FRET-APB. The FRET efficiency between D14 and SMXL7 peaked within 5 min of GR24^{5DS} treatment. Similar FRET efficiencies in the presence and absence of GR24^{5DS} were observed between d14^{seto} and SMXL7 (Figure 3H).

Together, these experiments indicate that D14 and SMAX1 can associate in the presence of GR24^{5DS}. Y2H and split-LUC experiments suggest that D14 can interact better with SMXL2 than with SMAX1, although this may be due, at least in part, to the instability of SMAX1 (Figure 3 and Supplemental Figure 7). The effect of d14^{seto} is less clear. Although Y2H experiments suggested that d14^{seto} was less able to interact with SMAX1, SMXL2, and SMXL7, this was not supported by split-LUC and FRET-APB assays in plants. The differences could be a consequence of overexpression or of the effects of other proteins in the plant cell environment (e.g., MAX2) on D14 signaling, interactions, and stability. Regardless, d14^{seto} was not as deficient as d14^{S97A} in its interactions with SMXL proteins or the GR24^{5DS} response, suggesting that it is hypomorphic rather than amorphic.

A hypomorphic d14 protein is more active when SMAX1 and SMXL2 are absent

Although D14 can induce degradation of SMAX1 and SMXL2, it is unclear whether this is only an artifact of treatments with an exogenous SL analog. If D14-mediated degradation of SMAX1 and SMXL2 has physiological significance, we can expect *d14* to affect growth processes controlled by SMAX1 and SMXL2 and/or *smax1 smxl2* to at least partially suppress *d14* phenotypes. As noted above, *d14-1* seedlings are phenotypically similar to the wild type. We found that *d14^{seto}* hypocotyls were slightly shorter than those of the wild type, suggesting that SMAX1/SMXL2 may be partially reduced (Supplemental Figure 8). However, *d14^{seto}* and *kai2 d14^{seto}* showed little response to GR24^{5DS}, implying that any such targeting by d14^{seto} may reflect promiscuous activity rather than an SL response, as suggested by the Y2H results (Figure 3A and Supplemental Figure 8).

We next examined the effects of *KAI2*, *SMAX1*, and *SMXL2* on the excess shoot-branching phenotype of *d14*. A recent study based on the overexpression of *SMAX1* proposed that SMAX1 suppresses axillary shoot branching (Zheng et al., 2021). Contrary to this result, we did not observe any effect of *kai2*,

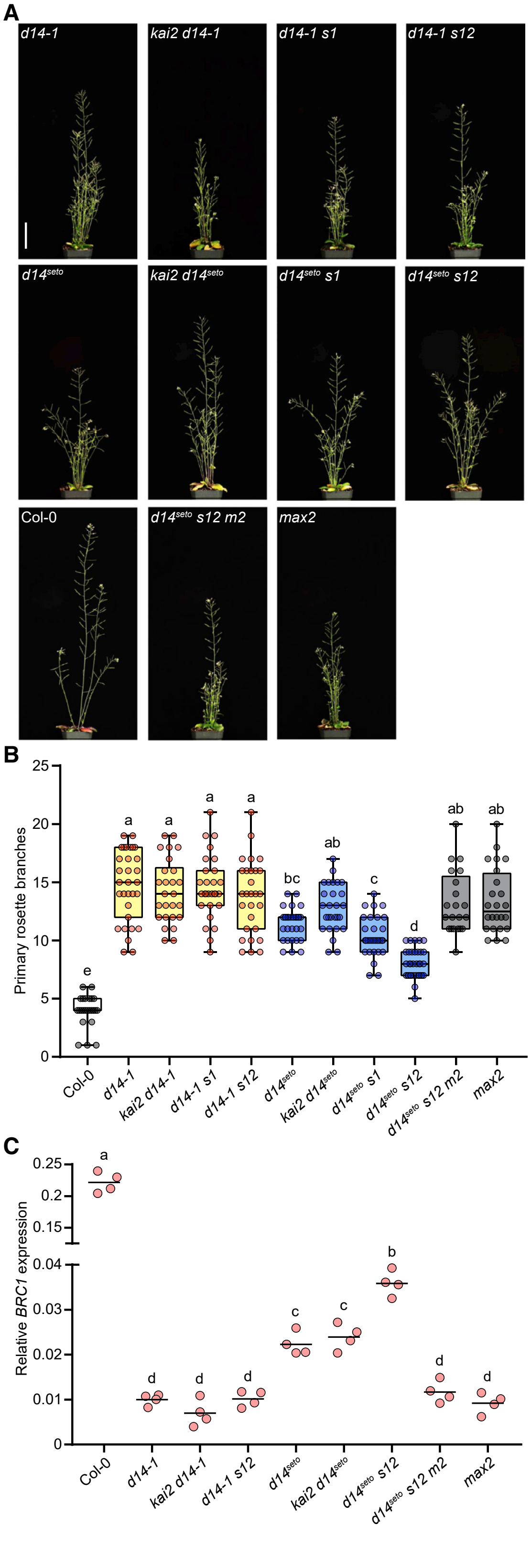

Figure 4. *d14^seto* is hypomorphic and more active in an *smax1 smxl2* background.

(A) Adult shoot morphology of Col-0, *d14-1*, *kai2 d14-1*, *d14-1 smax1*, *d14-1 smax1 smxl2*, *d14^seto*, *kai2 d14^seto*, *d14^seto smax1*, *d14^seto smax1 smxl2*, *d14^seto smax1 smxl2 max2*, and *max2* plants. Bar, 5 cm.

(B) The number of primary rosette branches of plant materials in (A). Box-and-whisker plots with the same letter are not significantly different from one another (Tukey's HSD, $p < 0.05$, n = 21–34).

(C) qRT-PCR analysis of *BRC1/TCP18* gene expression in non-elongated axillary buds of Col-0, *d14-1*, *kai2 d14-1*, *d14-1 smax1 smxl2*, *d14^seto*, *kai2 d14^seto*, *d14^seto smax1 smxl2*, *d14^seto smax1 smxl2 max2*, and *max2* plants collected 10 days after anthesis. Expression of *BRC1* is relative to the *CACS* internal reference gene. Scatter dot plots with the same letter are not significantly different from one another (bar indicates mean; n = 4 pooled tissue samples, three plants per pool; Student's t test, $p < 0.05$).

which overaccumulates SMAX1 and SMXL2, or *smax1 smxl2* on the excess branching phenotype of *d14-1* (Figures 4A and 4B). We also investigated genetic interactions between *d14^seto* and KAR signaling mutants. The excess branching phenotype of *d14^seto* was weaker than that of *d14-1*, consistent with d14^seto causing a partial loss of function. Interestingly, branching number was increased to *d14-1* and *max2* levels in the *kai2 d14^seto* mutant and reduced in *d14^seto smax1 smxl2*. Because *max2* was epistatic in the *d14^seto smax1 smxl2 max2* mutant, SMAX1 and SMXL2 are unlikely to regulate shoot branching downstream of MAX2. Instead, these data suggest that SMAX1 and SMXL2 negatively affect the ability of d14^seto to target SMXL6, SMXL7, and SMXL8 for degradation.

We found further support for this idea from analysis of *BRANCHED1* (*BRC1*) expression in non-elongated axillary buds. BRC1 is a transcription factor that represses axillary bud outgrowth and whose expression is negatively regulated by SMXL6, SMXL7, and SMXL8 (Aguilar-Martínez et al., 2007; Soundappan et al., 2015; Wang et al., 2020a). Consistent with the shoot-branching data, *smax1 smxl2* did not increase *BRC1* expression in the *d14-1* background. *BRC1* expression was higher in *d14^seto* buds than in *d14-1* buds, and the addition of *smax1 smxl2* mutations further increased *BRC1* expression in a *MAX2*-dependent manner (Figure 4C).

SMAX1 and SMXL2 may enhance D14 turnover after SL perception

One way that SMAX1 and SMXL2 might affect the activity of d14^seto is by reducing its abundance. D14 and KAI2 are both degraded within hours after activation (Chevalier et al., 2014; Waters et al., 2015b; Hu et al., 2017). KAI2 degradation after KAR treatment is MAX2-independent and probably occurs through association with SMAX1 and SMXL2, which are unstable (Waters et al., 2015b; Khosla et al., 2020b). D14 degradation after GR24 treatment is MAX2-dependent in Arabidopsis (Chevalier et al., 2014). If d14^seto is more prone to interactions with SMAX1 (Figures 3A and 3C), however, it may undergo increased turnover compared with wild-type D14. This led us to test the degradation of D14-GFP and d14^seto-GFP fusions expressed in wild-type seedlings after treatment with *rac*-GR24. We observed a faster rate of decline for d14^seto-GFP than for D14-GFP in both hypocotyl and root tissues of seedlings after *rac*-GR24 treatment (Figure 5A).

To assess whether SMAX1 and SMXL2 influence GR24^5DS-induced degradation of D14, we next introduced a *UBQ:D14-LUC* transgene into wild-type and *smax1 smxl2* backgrounds. The decline in bioluminescence from D14-LUC following GR24^5DS treatment was slowed in *smax1 smxl2* at all time points

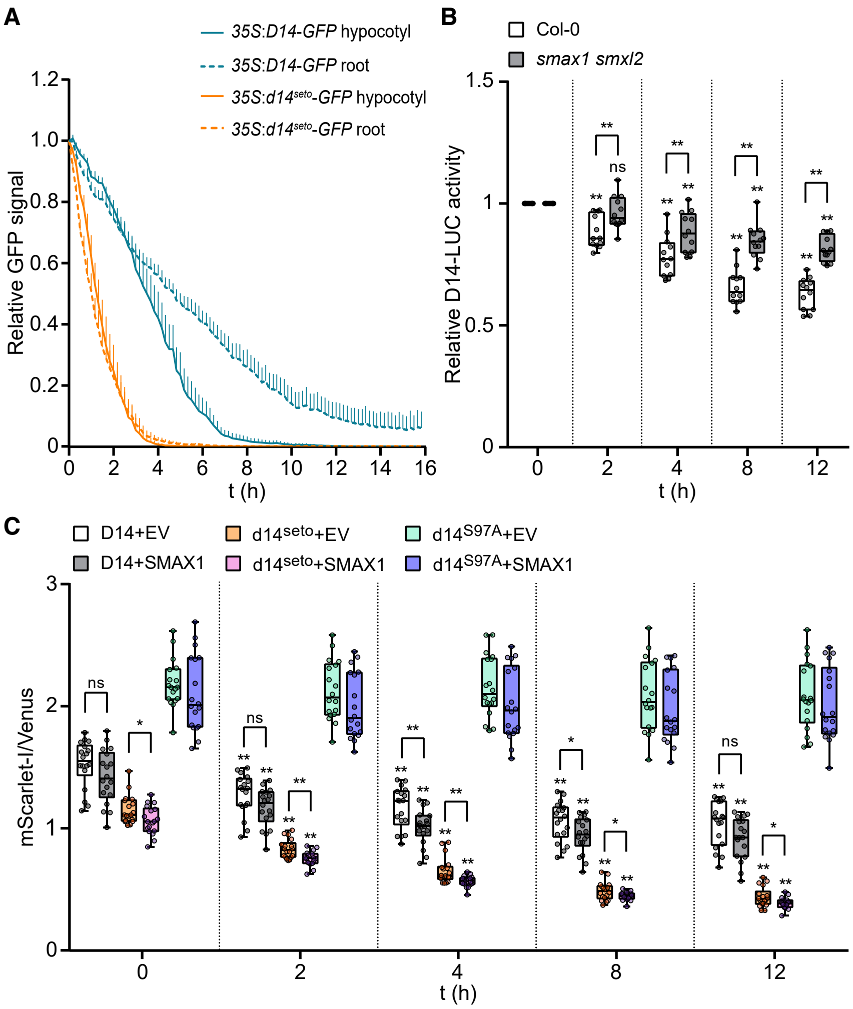

Figure 5. D14 degradation after GR24^{5DS} treatment is enhanced by *SMAX1* and *SMXL2*.

(A) The relative GFP signal from *D14-GFP* or *d14^{seto}-GFP* transgenic plants was measured every 10 min in the presence of 5 μM *rac*-GR24. The curve was generated from the mean value per genotype/treatment at each time point. Bar indicates SE of the mean (n = 6 seedlings).

(B) *UBQ:D14-LUC* transgenic seedlings in the Col-0 and *smax1 smxl2* backgrounds were treated with 5 μM GR24^{5DS} or acetone for 12 h. Bioluminescence is shown as relative LUC activity at 0, 2, 4, 8, and 12 h after treatment. n = 10–12 seedlings. Asterisks indicate significant differences relative to each group at 0 h or between compared pairs using Student's t test (*$p < 0.05$ and **$p < 0.01$; ns, no significance).

(C) Time-course assay of D14, d14^{seto}, and d14^{S97A} stability in *N. benthamiana* under 10 μM GR24^{5DS} treatment. Relative fluorescence from the D14-mScarlet-I reporter, the d14^{seto}-mScarlet-I reporter, or the d14^{S97A}-mScarlet-I reporter and the Venus reference after transient co-expression of the ratiometric system and SMAX1 effector in tobacco is shown. Leaf discs were treated for 12 h to monitor D14, d14^{seto}, and d14^{S97A} stability. n = 14 leaf discs. Asterisks indicate significant differences relative to each group at 0 h or between compared pairs using Student's t test (*$p < 0.05$ and **$p < 0.01$; ns, no significance).

compared with the wild type, suggesting that D14-LUC was partially stabilized by the absence of SMAX1 and SMXL2 (Figure 5B). We then transiently expressed *D14*, *d14^{seto}*, and *d14^{S97A}* ratiometric reporters with or without Arabidopsis SMAX1 in *Nbd14* leaves (Figure 5C). The d14^{S97A} reporter was the most stable of the three variants; it showed the highest relative abundance and was unaffected by GR24^{5DS} treatment. D14 and d14^{seto} reporters both declined in the 12 h after GR24^{5DS} treatment. As in Arabidopsis, d14^{seto} showed a faster rate of decline in tobacco. Co-expression of *SMAX1* caused a small but significant increase in GR24^{5DS}-induced turnover of D14 at two time points and of d14^{seto} at all time points. This suggested that the interaction of D14 with SMAX1 and SMXL2 may reduce its abundance in the presence of GR24; increased availability of a partially active d14^{seto} protein may explain why the *d14^{seto} smax1 smxl2* mutant showed partially recovered shoot branching.

D14-SCF^{MAX2} mediates SMAX1 degradation induced by osmotic stress

Although D14 and KAI2 often affect different developmental traits, this is not always the case. For example, both the SL and KAR/KL pathways promote drought tolerance in Arabidopsis (Li et al., 2017, 2020a, 2020b; Haider et al., 2018). Our data suggest that D14 has no effect on SMAX1 and SMXL2 degradation in response to endogenous SLs during seedling photomorphogenesis or shoot branching. We reasoned that D14-mediated degradation of SMAX1 and SMXL2 might be physiologically relevant for some traits regulated by both pathways or under conditions in which endogenous SL levels are sufficiently high. SL biosynthesis genes are induced by dehydration or mild drought in Arabidopsis and rice, leading to increased SL, at least in rice roots (Van Ha et al., 2014; Haider et al., 2018).

Therefore, as an alternative means of imposing drought/water deficit, we examined the response of KAR and SL signaling pathway mutants to osmotic stress. Wild-type seedlings grown in the presence of 300 mM mannitol showed a 40% reduction in fresh weight compared with seedlings grown on standard medium (Figures 6A and 6B). Growth inhibition by mannitol was enhanced in *d14* and *kai2* seedlings, and mannitol also caused a reduction in chlorophyll content (Figures 6A and 6C). We found that *smxl6,7,8* seedlings were even more strongly affected by mannitol than *kai2* and *d14*. By contrast, *smax1 smxl2* seedlings were resistant to mannitol, showing only a 10% reduction in fresh weight and an increase in chlorophyll content under mannitol treatment. Intriguingly, *SMAX1* and *SMXL2* contributed differently to osmotic stress tolerance. Under mannitol treatment, we observed less reduction in biomass in *smax1* seedlings and higher chlorophyll content in *smxl2* seedlings compared with the wild type (Supplemental Figure 9).

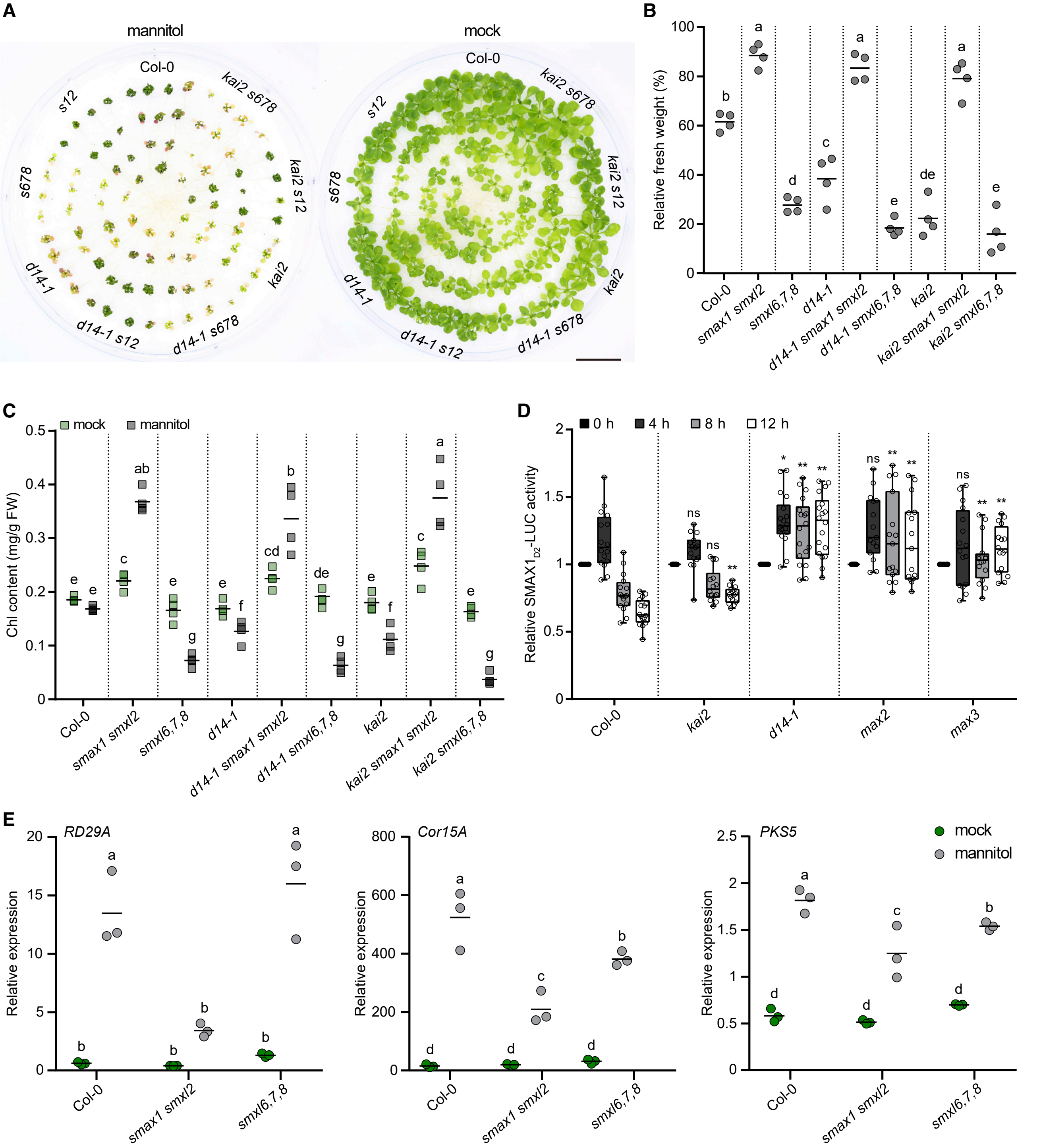

Figure 6. D14 targets SMAX1 and SMXL2 under osmotic stress.

(A) Twenty-one-day-old seedlings of Col-0, *smax1 smxl2*, *smxl 6,7,8*, *d14-1*, *d14-1 smax1 smxl2*, *d14-1 smxl6,7,8*, *kai2*, *kai2 smax1 smxl2*, and *kai2 smxl6,7,8* grown under mock or 300 mM mannitol conditions for 14 days. Bar, 2 cm.

(B) Relative fresh weights of plant materials used in **(A)** after application of 300 mM mannitol. The weights of aerial parts from plants grown on 0.5× MS agar medium with 300 mM mannitol are scaled to those from plants grown on 0.5× MS agar medium. Scatter dot plots with the same letter are not significantly different from one another (bar indicates mean; n = 4; Student's t test, $p < 0.05$).

(C) Chlorophyll (Chl) contents in the aerial parts of Arabidopsis seedlings used in **(A)**. Others are as in **(B)**.

(legend continued on next page)

To assess the effect of the *smax1 smxl2* or *smxl6*,*7*,*8* mutant on osmotic-stress-induced gene expression, we performed quantitative RT-PCR of *RD29A*, *Cor15A*, and *PKS5* (Fujii et al., 2011; Liu et al., 2014). Induction of *RD29A*, *Cor15A*, and *PKS5* transcripts in response to mannitol treatment was impaired in *smax1 smxl2* seedlings. By comparison, *RD29A* showed normal upregulation in response to mannitol treatment in *smxl6*,*7*,*8* seedlings. *Cor15A* and *PKS5* were not as highly induced by mannitol in *smxl6,7,8* seedlings than in the wild type but were more highly induced than in *smax1 smxl2* (Figure 6E).

Because *smax1 smxl2* had phenotypes opposite to those of *d14* and *kai2* and was epistatic to both, we hypothesized that D14 might contribute to SMAX1 and SMXL2 degradation during mannitol treatment. To test this possibility, we compared degradation of the $SMAX1_{D2}$-LUC reporter after mannitol treatment in Col-0, *kai2, d14-1*, *max2*, and the SL biosynthetic mutant *max3*. We observed degradation of $SMAX1_{D2}$-LUC within 8 h of mannitol treatment in wild-type and *kai2* seedlings but not in *d14-1*, *max2*, or SL-deficient *max3* seedlings (Figure 6D). SMXL7-LUC was also destabilized in a *D14*-dependent manner under mannitol treatment, supporting the idea that the level of endogenous SL and/or D14-SCF^{MAX2} signaling is induced by osmotic stress (Supplemental Figure 10). These results suggest that SL-induced degradation of SMAX1 and SMXL2 via D14-SCF^{MAX2} is not just an artificial consequence of GR24 application but can also occur under specific environmental conditions.

DISCUSSION

Although there are strong similarities between the KAR/KL and SL signaling pathways, genetic and biochemical studies have suggested that they are well insulated by specific receptor-target interactions, enabling distinct developmental responses to KAR/KL and SL (Soundappan et al., 2015; Villaécija-Aguilar et al., 2019). Contradicting this model, here we have shown that D14 can target SMAX1 for degradation after SL analog treatments. Genetic tests indicated that SMAX1 and, to a lesser degree, SMXL2 regulate hypocotyl elongation, but SMXL6, SMXL7, and SMXL8 do not (Figure 1). This result implied that the D14-mediated effect of GR24 on hypocotyl elongation is due to D14-SMAX1 crosstalk. This idea was supported by the observation that an SMAX1 ratiometric reporter was degraded in *N. benthamiana* after $GR24^{5DS}$ treatment in a partially D14-dependent manner (Figure 2A). $GR24^{5DS}$-induced degradation of an $SMAX1_{D2}$ reporter in *Arabidopsis thaliana* was also blocked in the *d14* background (Figure 2G). Physical interactions between D14 and SMAX1, however, are weak at best (Figure 3). SMXL proteins, which are distantly related to HSP101 heat-shock proteins that form hexamers, may form multimeric complexes (Khosla et al., 2020b). If heterogeneous complexes form (e.g., composed of SMAX1 and non-SMAX1 subunits), it is possible that SMAX1 could be indirectly targeted for proteolysis by a non-cognate receptor (i.e., D14) that interacts with SMXL2 or SMXL7. However, SMAX1 degradation by D14 does not require the presence of SMXL2, as demonstrated by the GR24 response of *kai2 smxl2* seedlings (Figure 2), nor does GR24-induced degradation of SMAX1 and SMXL2 by D14 require SMXL6, SMXL7, or SMXL8, as shown by *kai2 smxl6,7,8* seedlings (Figure 1C).

Therefore, our data suggest that a direct interaction between D14-SCF^{MAX2} and SMAX1 can occur when an SL analog is applied. Similarly, D14 can crosstalk with SMXL2 in the presence of SL analogs (Wang et al., 2020b). By contrast, there is no indication that KAR application can cause KAI2-SCF^{MAX2} to target D53 or SMXL7 for degradation, and the current genetic evidence for such crosstalk is controversial (Jiang et al., 2013; Wang et al., 2015; Swarbreck et al., 2019; Villaécija-Aguilar et al., 2019; Khosla et al., 2020b). We propose an update of the fully insulated KAR/KL and SL signaling models to include one-way promiscuity, in which D14 crosstalk with SMAX1 and SMXL2 is a putative remnant of its evolution from a KAI2 paralog (see below; Figure 7).

SMAX1 can be targeted by D14 in Arabidopsis, but less well than SMXL2

It is likely that D14 has lower affinity for SMAX1 than for SMXL2. Although both SMAX1 and SMXL2 are able to co-immunoprecipitate KAI2 from Arabidopsis protoplasts in the presence of an agonist, only SMXL2 is effective at co-immunoprecipitation of D14 (Wang et al., 2020b). SMAX1 also did not interact with D14 *in vitro* in a pull-down assay (Yao et al., 2017). Likewise, we observed weaker Y2H interactions between D14 and SMAX1 than between D14 and SMXL2 (Figure 3A). In addition, we saw negligible luminescence in split-LUC assays for D14-SMAX1 interactions compared with D14-SMXL2 or D14-SMXL7. This may be due to MAX2-dependent and/or -independent degradation of SMAX1 that causes high turnover (Khosla et al., 2020b). The luminescence signal was increased in split-LUC assays between D14 and a degradation-resistant $SMAX1^{\Delta RGKT}$ mutant protein, although it was still weaker than that produced by D14-SMXL2 interactions (Figure 3 and Supplemental Figure 6). Finally, we note that although we observed a strong effect of 500 nM $GR24^{5DS}$ on hypocotyl elongation of both *smax1* and *smxl2*, , 2020b) observed different D14-mediated responses to 100 nM $GR24^{4DO}$ treatments in these mutants (Figure 1 and Supplemental Figure 3). The 100 nM $GR24^{4DO}$ treatment had only a small effect on hypocotyl elongation of *smxl2* but had a large effect on *smax1* seedlings, implying that SMAX1 may be less effectively degraded than SMXL2. Lower concentrations of SL may be required to induce D14 crosstalk with SMXL2 than with SMAX1. For developmental processes such as root-hair elongation, in which SMXL2 has a more prominent role than SMAX1, or root skewing, to which SMXL2 and SMAX1 contribute non-redundantly, endogenous SLs may be more likely to have an

(D) Bioluminescence of $SMAX1_{D2}$-LUC in Col-0, *kai2*, *d14-1*, *max2*, and *max3* backgrounds. Seedlings were treated with 300 mM mannitol or water (control) for 12 h. Bioluminescence is shown as relative LUC activity at 0, 4, 8, and 12 h after treatment. n = 16–18 seedlings. ns, no significance. $^{*}p < 0.05$, $^{**}p < 0.01$, Student's t test comparisons with the Col-0 control at each time point.

(E) Expression of *RD29A*, *Cor15A*, and *PKS5* relative to the *CACS* internal reference in Col-0, *smax1 smxl2*, and *smxl6*,*7*,*8* grown for 7 days under a long-day photoperiod (16 h light/8 h dark) after 3 h mock or 300 mM mannitol treatment. Scatter dot plots with the same letter are not significantly different from one another (bar indicates mean; n = 3; Student's t test, $p < 0.05$).

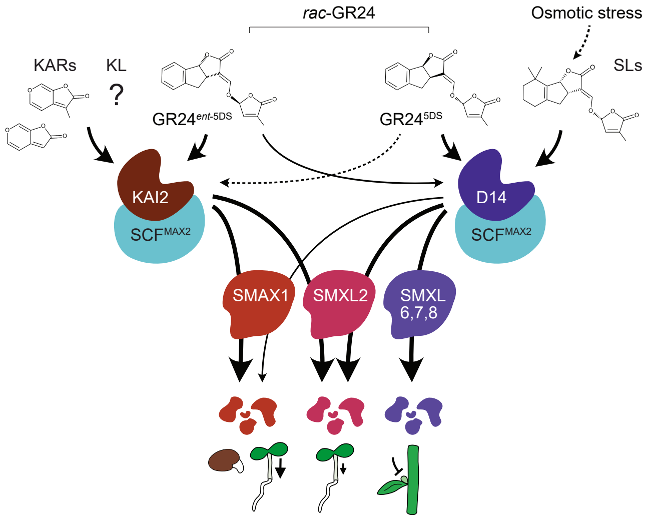

Figure 7. Model for crosstalk between SL and KAR/KL signaling pathways.

KAI2 recruits the SCF^{MAX2} E3 ubiquitin ligase complex upon the perception of KAR/KL or $GR24^{ent-5DS}$ to target SMAX1 and SMXL2 for degradation. SL or $GR24^{5DS}$ induces association of D14 with SCF^{MAX2} and SMXL7, SMXL2, and, to a lesser extent, SMAX1. This subsequently causes MAX2-dependent degradation of the targets. $GR24^{ent-5DS}$ activates D14 more weakly than $GR24^{5DS}$. $GR24^{5DS}$ may trigger KAI2 signaling to a limited degree (dotted line), although evidence of ligand-binding and *in vitro* activation is missing. Degradation of SMXL7 represses shoot branching, whereas degradation of SMAX1 represses seed germination and hypocotyl elongation. SMXL2 plays a minor role in hypocotyl elongation compared with SMAX1. In seedlings, endogenous SL is insufficient to trigger crosstalk between D14 and SMAX1. It occurs, however, in the presence of GR24 and under some conditions, such as osmotic stress, that may raise SL levels.

effect via D14-mediated crosstalk (Villaécija-Aguilar et al., 2019). It is currently unknown whether D14 can crosstalk with SMAX1 orthologs in other species. At least in rice, OsSMAX1 (LOC_Os08g15230) does not appear to be an interaction partner or target of D14 (Zheng et al., 2020).

Evolution of target preferences in KAR/KL and SL signaling pathways

Regardless of whether non-cognate interactions between D14 and SMAX1/SMXL2 affect development under physiological conditions, it is clear that the cognate interactions between D14 and D53-type SMXL proteins are important for SL-regulated growth in plants. This raises the question of how D14 and D53-type SMXL proteins evolved a specificity in their interactions that largely prevents crosstalk between the homologous SL and KAR/KL pathways in angiosperms. SLs have ancient origins in the land plant lineage (Yoneyama et al., 2018; Walker et al., 2019). However, D14 orthologs are observed only in the seed-bearing lineage (gymnosperms and angiosperms) (Bythell-Douglas et al., 2017). Gymnosperms have putative SMAX1 orthologs, but D53 orthologs are only found in angiosperms (Walker et al., 2019). Thus, the canonical D14-SCF^{MAX2}-D53 SL signaling mechanism is a feature of angiosperms. An attractive hypothesis, however, is that KAI2-like proteins function as SL receptors that target SMAX1 for degradation in other land plants. This is quite plausible given that such a mechanism is used by the seeds of obligate parasitic plants in the Orobanchaceae to sense host-derived SLs and germinate (Nelson, 2021).

One way that selective protein-protein interactions between KAI2-SMAX1 and D14-SMXL7 could have evolved is via mutations in an SL-responsive KAI2 paralog (a proto-D14) that disrupt SMAX1 interactions, combined with compensatory mutations in an SMAX1 paralog (a proto-SMXL7) that establish an orthogonal interaction with the proto-D14. However, this evolutionary path involves an intermediate phase during which the proto-D14 is a pseudogene and/or the proto-SMXL7 is misregulated, with potentially detrimental effects. Bacterial toxin-antitoxin systems have revealed an alternative way in which duplicated protein pairs may evolve selective interactions: via a promiscuous intermediate state (Aakre et al., 2015). According to a promiscuity-based model, proto-D14 might first acquire a mutation that broadens its potential interaction specificity. This would enable proto-SMXL7 to acquire a mutation that blocks interactions with KAI2 but maintains interactions with proto-D14, without negatively affecting fitness. Subsequently, proto-D14 may acquire another mutation that narrows its interaction specificity to proto-SMXL7 alone. Throughout this process, SMXL7 regulation would continue. Substantial work will be needed to evaluate this hypothesis. However, we propose that the ability of D14 to engage in a non-preferred interaction with SMAX1 could be a remnant of such an evolutionary process.

Effects of the $d14^{seto}$ allele

The $d14^{seto}$ allele, which causes a Pro169Leu substitution, appears to reduce the selectivity of D14 against SMAX1 interactions (Figures 3A and 3C). Pro169 is a highly conserved (>90%) surface residue found within a small motif that distinguishes D14 and KAI2 proteins (ADV—P versus GDMDS, respectively) (Chevalier et al., 2014; Bythell-Douglas et al., 2017). As such, it has been hypothesized to be a specificity-determining position (Chevalier et al., 2014). Alternatively, this motif may influence SL perception. The motif containing Pro169 comprises most of a short loop that joins the αT2 and αT3 helices (also known as αE and αF) of D14. The composition of this loop affects the rigidity of the ligand-binding pocket, which in turn affects ligand affinities (Bürger et al., 2019).

Our results suggested that *$d14^{seto}$* causes a partial loss of function in SL signaling, as it had weaker branching and leaf morphology phenotypes than the null T-DNA insertion allele *d14-1* (Figure 4B and Supplemental Figure 11). This result implied that $d14^{seto}$ was less effective at triggering SL-induced degradation of SMXL6, SMXL7, and SMXL8. However, in transient expression experiments in *N. benthamiana*, $d14^{seto}$ showed an ability similar to that of wild-type D14 to interact with SMXL7 and cause its degradation (Figures 2E, 3B, 3F, and 3H). Therefore, we hypothesized that the $d14^{seto}$ protein may have reduced function because of higher instability. Supporting this notion, we found that $d14^{seto}$ was more rapidly degraded following $GR24^{5DS}$ treatment than wild-type D14 in Arabidopsis and *N. benthamiana* (Figures 5A and 5C).

KAI2 degradation after KAR treatment is MAX2-independent and is probably driven by association with unstable SMAX1 and/or SMXL2 proteins (Waters et al., 2015b; Khosla et al., 2020b). We

found that D14 instability was reduced in the *smax1 smxl2* background (Figure 5B), suggesting that it may also be degraded by association with SMAX1 and/or SMXL2. This led us to hypothesize that enhanced d14^{seto} turnover after SL perception might be caused by stronger association with SMAX1 and/or SMXL2 compared with wild-type D14. Indeed, co-expression of SMAX1 slightly enhanced d14^{seto} degradation in *N. benthamiana* (Figure 5C). This hypothesis also predicts that the phenotypes of *d14^{seto}* will be affected by SMAX1/SMXL2 abundance. Consistent with this prediction, the branching phenotype of *d14^{seto}* was increased by the addition of *kai2*. Overaccumulation of SMAX1 and SMXL2 in *kai2* might further reduce d14^{seto} abundance (Figure 4B). Conversely, the excess branching of *d14^{seto}* was partially suppressed by *smax1 smxl2*, perhaps indicating that the d14^{seto} protein had been stabilized. Similarly, *smax1 smxl2* partially suppressed the reduced *BRC1* expression in *d14^{seto}* (Figures 4B and 4C). By comparison, *smax1 smxl2* had no effect on branching or *BRC1* expression in the null *d14-1* background (Figures 4B and 4C).

The physiological relevance of D14-SMAX1 crosstalk

Although D14 can target SMAX1 and SMXL2 for degradation after SL treatment, SL-deficient and SL-insensitive mutants do not show phenotypes associated with SMAX1 and SMXL2 overaccumulation, suggesting that this crosstalk does not normally occur (Nelson et al., 2011; Waters et al., 2012; Soundappan et al., 2015). Alternatively, SL levels that are sufficiently high to stimulate D14 crosstalk may occur only in limited developmental contexts. SL biosynthesis is induced by various stresses such as drought and phosphate starvation (López-Ráez et al., 2008; Van Ha et al., 2014; Haider et al., 2018). This led us to explore whether D14-SMAX1 crosstalk occurs during water stress. Interestingly, although *smxl6,7,8* plants have enhanced resistance to water deficit, opposite to *d14* and SL-deficient mutants, we found that *smxl6,7,8* seedlings are more susceptible to osmotic stress (Van Ha et al., 2014; Li et al., 2020a, 2020b) (Figures 6A–6C). This was particularly surprising because *d14* was also more susceptible to osmotic stress than the wild type. By contrast, *smax1 smxl2* had enhanced resistance to osmotic stress and was epistatic to *d14* and *kai2* for this trait (Figures 6A–6C). Defective induction of *RD29A* and *Cor15A* expression in *smax1 smxl2* may confer osmotic stress tolerance by strengthening photosynthesis and seedling growth (Msanne et al., 2011; Liu et al., 2014). Alternatively, given that *smax1 smxl2* seedlings showed better growth under mannitol treatment than the wild type, the reduced upregulation of *RD29A*, *Cor15A*, and *PKS5* by mannitol may indicate that *smax1 smxl2* is less susceptible to osmotic stress. Although we cannot yet explain *smxl6,7,8* phenotypes, this result suggested that D14 might target SMAX1 and SMXL2 under osmotic stress. Indeed, we observed enhanced degradation of an $SMAX1_{D2}$ reporter following osmotic stress—without GR24 treatments—that was dependent on *D14* and the SL biosynthesis gene *MAX3*. It is also notable that KAR-responsive genes are upregulated under osmotic stress (Shah et al., 2020). This implies a reduction in SMAX1 and SMXL2 levels, which could potentially be due to SL signaling activity. In conclusion, we propose that under some environmental conditions or developmental contexts, D14 crosstalk initiated by SLs may broaden the ability of plants to fine-tune SMAX1 and SMXL2 regulation.

METHODS

Plant materials

The *Arabidopsis thaliana* mutants *d14-1*, *d14^{seto}*, *htl-3* (a *kai2* allele), *d14-1 htl-3*, *max2-1*, *smax1-2*, *smxl2-1*, *smax1-2 smxl2-1*, *smxl6-4 smxl7-3 smxl8-1*, and *max3-11* have been described previously (Waters et al., 2012; Stanga et al., 2013, 2016; Chevalier et al., 2014; Toh et al., 2014; Soundappan et al., 2015). All lines are in the Col-0 ecotype. Genotyping primers are listed in Supplemental Table 1. Detailed methods are found in the supplemental information.

SUPPLEMENTAL INFORMATION

Supplemental information is available at *Plant Communications Online*.

FUNDING

We gratefully acknowledge funding support from US National Science Foundation (NSF) grants IOS-1737153, IOS-1740560, and IOS-1856741 to D.C.N. and Spanish Ministry of Science and Innovation grants BIO2017-84363-R and PID2020-112779RB-I00 and FESF Investing in Your Future to P.C.

AUTHOR CONTRIBUTIONS

Experiments were designed, carried out, and analyzed by Q.L., E.S.M.-F., A.K., A.R.F.W., and S.C. Figures were prepared by Q.L. The manuscript was prepared by Q.L. and D.C.N. with contributions and final approval from all authors. The project was designed by Q.L., E.S.M.-F., P.C., and D.C.N. Funding to support the project was secured by P.C. and D.C.N.

ACKNOWLEDGMENTS

We thank Dr. Gavin Flematti and Dr. Adrian Scaffidi (University of Western Australia) for supplying KAR_1, KAR_2, *rac*-GR24, and purified GR24 enantiomers. No conflict of interest declared.

Received: August 3, 2021
Revised: December 15, 2021
Accepted: January 25, 2022
Published: January 31, 2022

SUPPORTING CITATIONS

The following references appear in the Supplemental Information: Gietz and Woods, 2002; Goddard-Borger et al., 2007.

REFERENCES

Aakre, C.D., Herrou, J., Phung, T.N., Perchuk, B.S., Crosson, S., and Laub, M.T. (2015). Evolving new protein-protein interaction specificity through promiscuous intermediates. Cell **163**:594–606.

Aguilar-Martínez, J.A., Poza-Carrión, C., and Cubas, P. (2007). Arabidopsis BRANCHED1 acts as an integrator of branching signals within axillary buds. Plant Cell **19**:458–472.

Agusti, J., Herold, S., Schwarz, M., Sanchez, P., Ljung, K., Dun, E.A., Brewer, P.B., Beveridge, C.A., Sieberer, T., Sehr, E.M., et al. (2011). Strigolactone signaling is required for auxin-dependent stimulation of secondary growth in plants. Proc. Natl. Acad. Sci. U S A **108**:20242–20247.

Akiyama, K., Matsuzaki, K.-I., and Hayashi, H. (2005). Plant sesquiterpenes induce hyphal branching in arbuscular mycorrhizal fungi. Nature **435**:824–827.

Blázquez, M.A., Nelson, D.C., and Weijers, D. (2020). Evolution of plant hormone response pathways. Annu. Rev. Plant Biol. **71**:327–353.

Bouwmeester, H., Li, C., Thiombiano, B., Rahimi, M., and Dong, L. (2021). Adaptation of the parasitic plant lifecycle: germination is controlled by essential host signaling molecules. Plant Physiol. **185**:1292–1308.

Bürger, M., Mashiguchi, K., Lee, H.J., Nakano, M., Takemoto, K., Seto, Y., Yamaguchi, S., and Chory, J. (2019). Structural basis of karrikin and non-natural strigolactone perception in physcomitrella patens. Cell Rep. **26**:855–865.e5.

Bythell-Douglas, R., Rothfels, C.J., Stevenson, D.W.D., Graham, S.W., Wong, G.K.-S., Nelson, D.C., and Bennett, T. (2017). Evolution of strigolactone receptors by gradual neo-functionalization of KAI2 paralogues. BMC Biol. **15**:52.

Carbonnel, S., Das, D., Varshney, K., Kolodziej, M.C., Villaécija-Aguilar, J.A., and Gutjahr, C. (2020a). The karrikin signaling regulator SMAX1 controls Lotus japonicus root and root hair development by suppressing ethylene biosynthesis. Proc. Natl. Acad. Sci. U S A **117**:21757–21765.

Carbonnel, S., Torabi, S., Griesmann, M., Bleek, E., Tang, Y., Buchka, S., Basso, V., Shindo, M., Boyer, F.-D., Wang, T.L., et al. (2020b). Lotus japonicus karrikin receptors display divergent ligand-binding specificities and organ-dependent redundancy. PLoS Genet. **16**:e1009249.

Chevalier, F., Nieminen, K., Sánchez-Ferrero, J.C., Rodríguez, M.L., Chagoyen, M., Hardtke, C.S., and Cubas, P. (2014). Strigolactone promotes degradation of DWARF14, an α/β hydrolase essential for strigolactone signaling in Arabidopsis. Plant Cell **26**:1134–1150.

Conn, C.E., and Nelson, D.C. (2015). Evidence that KARRIKIN-INSENSITIVE2 (KAI2) receptors may perceive an unknown signal that is not karrikin or strigolactone. Front. Plant Sci. **6**:1219.

Cook, C.E., Whichard, L.P., Turner, B., Wall, M.E., and Egley, G.H. (1966). Germination of witchweed (Striga lutea Lour.): isolation and properties of a potent stimulant. Science **154**:1189–1190.

Day, R.N., Periasamy, A., and Schaufele, F. (2001). Fluorescence resonance energy transfer microscopy of localized protein interactions in the living cell nucleus. Methods **25**:4–18.

de Saint Germain, A., Clavé, G., Badet-Denisot, M.-A., Pillot, J.-P., Cornu, D., Le Caer, J.-P., Burger, M., Pelissier, F., Retailleau, P., Turnbull, C., et al. (2016). An histidine covalent receptor and butenolide complex mediates strigolactone perception. Nat. Chem. Biol. **12**:787–794.

Flematti, G.R., Ghisalberti, E.L., Dixon, K.W., and Trengove, R.D. (2004). A compound from smoke that promotes seed germination. Science **305**:977.

Flematti, G.R., Scaffidi, A., Waters, M.T., and Smith, S.M. (2016). Stereospecificity in strigolactone biosynthesis and perception. Planta **243**:1361–1373.

Fujii, H., Verslues, P.E., and Zhu, J.-K. (2011). Arabidopsis decuple mutant reveals the importance of SnRK2 kinases in osmotic stress responses in vivo. Proc. Natl. Acad. Sci. U S A **108**:1717–1722.

Gietz, R.D., and Woods, R.A. (2002). Transformation of yeast by lithium acetate/single-stranded carrier DNA/polyethylene glycol method. Methods Enzymol. **350**:87–96.

Goddard-Borger, E.D., Ghisalberti, E.L., and Stick, R.V. (2007). Synthesis of the germination stimulant 3-methyl-2H-furo[2,3-c]pyran-2-one and analogous compounds from carbohydrates. Eur. J. Org. Chem. **38**:3925–3934.

Gomez-Roldan, V., Fermas, S., Brewer, P.B., Puech-Pagès, V., Dun, E.A., Pillot, J.-P., Letisse, F., Matusova, R., Danoun, S., Portais, J.-C., et al. (2008). Strigolactone inhibition of shoot branching. Nature **455**:189–194.

Haider, I., Andreo-Jimenez, B., Bruno, M., Bimbo, A., Floková, K., Abuauf, H., Ntui, V.O., Guo, X., Charnikhova, T., Al-Babili, S., et al. (2018). The interaction of strigolactones with abscisic acid during the drought response in rice. J. Exp. Bot. **69**:2403–2414.

Hamiaux, C., Drummond, R.S.M., Janssen, B.J., Ledger, S.E., Cooney, J.M., Newcomb, R.D., and Snowden, K.C. (2012). DAD2 is an α/β hydrolase likely to be involved in the perception of the plant branching hormone, strigolactone. Curr. Biol. **22**:2032–2036.

Hu, Q., He, Y., Wang, L., Liu, S., Meng, X., Liu, G., Jing, Y., Chen, M., Song, X., Jiang, L., et al. (2017). DWARF14, A receptor covalently linked with the active form of strigolactones, undergoes strigolactone-dependent degradation in rice. Front. Plant Sci. **8**:1935.

Jain, N., Kulkarni, M.G., and van Staden, J. (2006). A butenolide, isolated from smoke, can overcome the detrimental effects of extreme temperatures during tomato seed germination. Plant Growth Regul. **49**:263–267.

Jiang, L., Liu, X., Xiong, G., Liu, H., Chen, F., Wang, L., Meng, X., Liu, G., Yu, H., Yuan, Y., et al. (2013). DWARF 53 acts as a repressor of strigolactone signalling in rice. Nature **504**:401–405.

Kalliola, M., Jakobson, L., Davidsson, P., Pennanen, V., Waszczak, C., Yarmolinsky, D., Zamora, O., Palva, E.T., Kariola, T., Kollist, H., et al. (2020). Differential role of MAX2 and strigolactones in pathogen, ozone, and stomatal responses. Plant Direct **4**:e00206.

Khosla, A., Rodriguez-Furlan, C., Kapoor, S., Van Norman, J.M., and Nelson, D.C (2020a). A series of dual-reporter vectors for ratiometric analysis of protein abundance in plants. Plant Direct **4**:e00231.

Khosla, A., Morffy, N., Li, Q., Faure, L., Chang, S.H., Yao, J., Zheng, J., Cai, M.L., Stanga, J., Flematti, G.R., et al. (2020b). Structure–function analysis of SMAX1 reveals domains that mediate its karrikin-induced proteolysis and interaction with the receptor KAI2. Plant Cell **32**:2639–2659.

Kobae, Y., Kameoka, H., Sugimura, Y., Saito, K., Ohtomo, R., Fujiwara, T., and Kyozuka, J. (2018). Strigolactone biosynthesis genes of rice are required for the punctual entry of arbuscular mycorrhizal fungi into the roots. Plant Cell Physiol. **59**:544–553.

Kochanek, J., Long, R.L., Lisle, A.T., and Flematti, G.R. (2016). Karrikins identified in biochars indicate post-fire chemical cues can influence community diversity and plant development. PLoS One **11**:e0161234.

Lahari, Z., Ullah, C., Kyndt, T., Gershenzon, J., and Gheysen, G. (2019). Strigolactones enhance root-knot nematode (Meloidogyne graminicola) infection in rice by antagonizing the jasmonate pathway. New Phytol. **224**:454–465.

Li, W., Nguyen, K.H., Chu, H.D., Van Ha, C., Watanabe, Y., Osakabe, Y., Leyva-González, M.A., Sato, M., Toyooka, K., Voges, L., et al. (2017). The karrikin receptor KAI2 promotes drought resistance in Arabidopsis thaliana. PLoS Genet. **13**:e1007076.

Li, W., Nguyen, K.H., Tran, C.D., Watanabe, Y., Tian, C., Yin, X., Li, K., Yang, Y., Guo, J., Miao, Y., et al. (2020a). Negative roles of strigolactone-related SMXL6, 7 and 8 proteins in drought resistance in Arabidopsis. Biomolecules **10**:607.

Li, W., Nguyen, K.H., Chu, H.D., Watanabe, Y., Osakabe, Y., Sato, M., Toyooka, K., Seo, M., Tian, L., Tian, C., et al. (2020b). Comparative functional analyses of DWARF14 and KARRIKIN INSENSITIVE 2 in drought adaptation of Arabidopsis thaliana. Plant J. **103**:111–127.

Liang, Y., Ward, S., Li, P., Bennett, T., and Leyser, O. (2016). SMAX1-LIKE7 signals from the nucleus to regulate shoot development in Arabidopsis via partially EAR motif-independent mechanisms. Plant Cell **28**:1581–1601.

Liu, D., Li, W., Cheng, J., and Hou, L. (2014). Expression analysis and functional characterization of a cold-responsive gene COR15A from Arabidopsis thaliana. Acta Physiol. Plant **36**:2421–2432.

López-Ráez, J.A., Charnikhova, T., Gómez-Roldán, V., Matusova, R., Kohlen, W., De Vos, R., Verstappen, F., Puech-Pages, V., Bécard, G., Mulder, P., et al. (2008). Tomato strigolactones are derived from carotenoids and their biosynthesis is promoted by phosphate starvation. New Phytol. **178**:863–874.

Msanne, J., Lin, J., Stone, J.M., and Awada, T. (2011). Characterization of abiotic stress-responsive Arabidopsis thaliana RD29A and RD29B genes and evaluation of transgenes. Planta **234**:97–107.

Nasir, F., Tian, L., Shi, S., Chang, C., Ma, L., Gao, Y., and Tian, C. (2019). Strigolactones positively regulate defense against Magnaporthe oryzae in rice (Oryza sativa). Plant Physiol. Biochem. **142**:106–116.

Nelson, D.C. (2021). The mechanism of host-induced germination in root parasitic plants. Plant Physiol. **185**:1353–1373.

Nelson, D.C., Flematti, G.R., Riseborough, J.-A., Ghisalberti, E.L., Dixon, K.W., and Smith, S.M. (2010). Karrikins enhance light responses during germination and seedling development in Arabidopsis thaliana. Proc. Natl. Acad. Sci. U S A **107**:7095–7100.

Nelson, D.C., Scaffidi, A., Dun, E.A., Waters, M.T., Flematti, G.R., Dixon, K.W., Beveridge, C.A., Ghisalberti, E.L., and Smith, S.M. (2011). F-box protein MAX2 has dual roles in karrikin and strigolactone signaling in Arabidopsis thaliana. Proc. Natl. Acad. Sci. U S A **108**:8897–8902.

Nelson, D.C., Flematti, G.R., Ghisalberti, E.L., Dixon, K.W., and Smith, S.M. (2012). Regulation of seed germination and seedling growth by chemical signals from burning vegetation. Annu. Rev. Plant Biol. **63**:107–130.

Rasmussen, A., Mason, M.G., De Cuyper, C., Brewer, P.B., Herold, S., Agusti, J., Geelen, D., Greb, T., Goormachtig, S., Beeckman, T., et al. (2012). Strigolactones suppress adventitious rooting in Arabidopsis and pea. Plant Physiol. **158**:1976–1987.

Scaffidi, A., Waters, M.T., Sun, Y.K., Skelton, B.W., Dixon, K.W., Ghisalberti, E.L., Flematti, G.R., and Smith, S.M. (2014). Strigolactone hormones and their stereoisomers signal through two related receptor proteins to induce different physiological responses in Arabidopsis. Plant Physiol. **165**:1221–1232.

Seto, Y., Yasui, R., Kameoka, H., Tamiru, M., Cao, M., Terauchi, R., Sakurada, A., Hirano, R., Kisugi, T., Hanada, A., et al. (2019). Strigolactone perception and deactivation by a hydrolase receptor DWARF14. Nat. Commun. **10**:191.

Shabek, N., Ticchiarelli, F., Mao, H., Hinds, T.R., Leyser, O., and Zheng, N. (2018). Structural plasticity of D3-D14 ubiquitin ligase in strigolactone signalling. Nature **563**:652–656.

Shah, F.A., Wei, X., Wang, Q., Liu, W., Wang, D., Yao, Y., Hu, H., Chen, X., Huang, S., Hou, J., et al. (2020). Karrikin improves osmotic and salt stress tolerance via the regulation of the redox homeostasis in the oil plant sapium sebiferum. Front. Plant Sci. **11**:216.

Soundappan, I., Bennett, T., Morffy, N., Liang, Y., Stanga, J.P., Abbas, A., Leyser, O., and Nelson, D.C. (2015). SMAX1-LIKE/D53 family members enable distinct MAX2-dependent responses to strigolactones and karrikins in Arabidopsis. Plant Cell **27**:3143–3159.

Stanga, J.P., Smith, S.M., Briggs, W.R., and Nelson, D.C. (2013). SUPPRESSOR OF MORE AXILLARY GROWTH2 1 controls seed germination and seedling development in Arabidopsis. Plant Physiol. **163**:318–330.

Stanga, J.P., Morffy, N., and Nelson, D.C. (2016). Functional redundancy in the control of seedling growth by the karrikin signaling pathway. Planta **243**:1397–1406.

Sun, X.-D., and Ni, M. (2011). HYPOSENSITIVE TO LIGHT, an alpha/beta fold protein, acts downstream of ELONGATED HYPOCOTYL 5 to regulate seedling de-etiolation. Mol. Plant **4**:116–126.

Swarbreck, S.M., Guerringue, Y., Matthus, E., Jamieson, F.J.C., and Davies, J.M. (2019). Impairment in karrikin but not strigolactone sensing enhances root skewing in Arabidopsis thaliana. Plant J. **98**:607–621.

Toh, S., Holbrook-Smith, D., Stokes, M.E., Tsuchiya, Y., and McCourt, P. (2014). Detection of parasitic plant suicide germination compounds using a high-throughput Arabidopsis HTL/KAI2 strigolactone perception system. Chem. Biol. **21**:988–998.

Ueda, H., and Kusaba, M. (2015). Strigolactone regulates leaf senescence in concert with ethylene in Arabidopsis. Plant Physiol. **169**:138–147.

Umehara, M., Hanada, A., Yoshida, S., Akiyama, K., Arite, T., Takeda-Kamiya, N., Magome, H., Kamiya, Y., Shirasu, K., Yoneyama, K., et al. (2008). Inhibition of shoot branching by new terpenoid plant hormones. Nature **455**:195–200.

Van Ha, C., Leyva-González, M.A., Osakabe, Y., Tran, U.T., Nishiyama, R., Watanabe, Y., Tanaka, M., Seki, M., Yamaguchi, S., Van Dong, N., et al. (2014). Positive regulatory role of strigolactone in plant responses to drought and salt stress. Proc. Natl. Acad. Sci. U S A **111**:851–856.

Villaécija-Aguilar, J.A., Hamon-Josse, M., Carbonnel, S., Kretschmar, A., Schmidt, C., Dawid, C., Bennett, T., and Gutjahr, C. (2019). SMAX1/SMXL2 regulate root and root hair development downstream of KAI2-mediated signalling in Arabidopsis. PLoS Genet. **15**:e1008327.

Walker, C.H., Siu-Ting, K., Taylor, A., O'Connell, M.J., and Bennett, T. (2019). Strigolactone synthesis is ancestral in land plants, but canonical strigolactone signalling is a flowering plant innovation. BMC Biol. **17**:70.

Wang, L., Wang, B., Jiang, L., Liu, X., Li, X., Lu, Z., Meng, X., Wang, Y., Smith, S.M., and Li, J. (2015). Strigolactone signaling in Arabidopsis regulates shoot development by targeting D53-like SMXL repressor proteins for ubiquitination and degradation. Plant Cell **27**:3128–3142.

Wang, L., Waters, M.T., and Smith, S.M. (2018). Karrikin-KAI2 signalling provides Arabidopsis seeds with tolerance to abiotic stress and inhibits germination under conditions unfavourable to seedling establishment. New Phytol. **219**:605–618.

Wang, L., Wang, B., Yu, H., Guo, H., Lin, T., Kou, L., Wang, A., Shao, N., Ma, H., Xiong, G., et al. (2020a). Transcriptional regulation of strigolactone signalling in Arabidopsis. Nature **583**:277–281.

Wang, L., Xu, Q., Yu, H., Ma, H., Li, X., Yang, J., Chu, J., Xie, Q., Wang, Y., Smith, S.M., et al. (2020b). Strigolactone and karrikin signaling pathways elicit ubiquitination and proteolysis of SMXL2 to regulate hypocotyl elongation in Arabidopsis. Plant Cell **32**:2251–2270.

Wang, Y., Yao, R., Du, X., Guo, L., Chen, L., Xie, D., and Smith, S.M. (2021). Molecular basis for high ligand sensitivity and selectivity of strigolactone receptors in Striga. Plant Physiol. **185**:1411–1428.

Waters, M.T., Nelson, D.C., Scaffidi, A., Flematti, G.R., Sun, Y.K., Dixon, K.W., and Smith, S.M. (2012). Specialisation within the DWARF14 protein family confers distinct responses to karrikins and strigolactones in Arabidopsis. Development **139**:1285–1295.

Waters, M.T., Scaffidi, A., Moulin, S.L.Y., Sun, Y.K., Flematti, G.R., and Smith, S.M. (2015a). A Selaginella moellendorffii ortholog of KARRIKIN INSENSITIVE2 functions in Arabidopsis development but cannot mediate responses to karrikins or strigolactones. Plant Cell **27**:1925–1944.

Waters, M.T., Scaffidi, A., Flematti, G., and Smith, S.M. (2015b). Substrate-induced degradation of the α/β-Fold hydrolase KARRIKIN INSENSITIVE2 requires a functional catalytic triad but is independent of MAX2. Mol. Plant **8**:814–817.

Waters, M.T., Gutjahr, C., Bennett, T., and Nelson, D.C. (2017). Strigolactone signaling and evolution. Annu. Rev. Plant Biol. **68**:291–322.

White, A.R.F., Mendez, J.A., Khosla, A., and Nelson, D.C. (2021). Rapid analysis of strigolactone receptor activity in a Nicotiana benthamiana dwarf14 mutant. Preprint at Biorxiv https://doi.org/10.1101/2021.05.11.443507.

Xu, Y., Miyakawa, T., Nosaki, S., Nakamura, A., Lyu, Y., Nakamura, H., Ohto, U., Ishida, H., Shimizu, T., Asami, T., et al. (2018). Structural

analysis of HTL and D14 proteins reveals the basis for ligand selectivity in Striga. Nat. Commun. **9**:3947.

Yamada, Y., Furusawa, S., Nagasaka, S., Shimomura, K., Yamaguchi, S., and Umehara, M. (2014). Strigolactone signaling regulates rice leaf senescence in response to a phosphate deficiency. Planta **240**:399–408.

Yao, R., Ming, Z., Yan, L., Li, S., Wang, F., Ma, S., Yu, C., Yang, M., Chen, L., Chen, L., et al. (2016). DWARF14 is a non-canonical hormone receptor for strigolactone. Nature **536**:469–473.

Yao, R., Wang, F., Ming, Z., Du, X., Chen, L., Wang, Y., Zhang, W., Deng, H., and Xie, D. (2017). ShHTL7 is a non-canonical receptor for strigolactones in root parasitic weeds. Cell Res. **27**:838–841.

Yao, J.R., Scaffidi, A., Meng, Y.J., Melville, K.T., Komatsu, A., Khosla, A., Nelson, D.C., Kyozuka, J., Flematti, G.R., and Waters, M.T. (2021). Desmethyl butenolides are optimal ligands for karrikin receptor proteins. New Phytol. **230**:1003–1016.

Yoneyama, K., Mori, N., Sato, T., Yoda, A., Xie, X., Okamoto, M., Iwanaga, M., Ohnishi, T., Nishiwaki, H., Asami, T., et al. (2018). Conversion of carlactone to carlactonoic acid is a conserved function ofMAX1 homologs in strigolactone biosynthesis. New Phytol. **218**:1522–1533.

Zheng, J., Hong, K., Zeng, L., Wang, L., Kang, S., Qu, M., Dai, J., Zou, L., Zhu, L., Tang, Z., et al. (2020). Karrikin signaling acts parallel to and additively with strigolactone signaling to regulate rice mesocotyl elongation in darkness. Plant Cell **32**:2780–2805.

Zheng, X., Yang, X., Chen, Z., Xie, W., Yue, X., Zhu, H., Chen, S., and Sun, X. (2021). Arabidopsis SMAX1 overaccumulation suppresses rosette shoot branching and promotes leaf and petiole elongation. Biochem. Biophys. Res. Commun. **553**:44–50.

Zhou, F., Lin, Q., Zhu, L., Ren, Y., Zhou, K., Shabek, N., Wu, F., Mao, H., Dong, W., Gan, L., et al. (2013). D14-SCF(D3)-dependent degradation of D53 regulates strigolactone signalling. Nature **504**:406–410.

Plant Communications, Volume 3

Supplemental information

The strigolactone receptor D14 targets SMAX1 for degradation in response to GR24 treatment and osmotic stress

Qingtian Li, Elena Sánchez Martín-Fontecha, Aashima Khosla, Alexandra R.F. White, Sunhyun Chang, Pilar Cubas, and David C. Nelson

Supplemental Information

The strigolactone receptor D14 targets SMAX1 for degradation in response to GR24 treatment and osmotic stress

Qingtian Li[1], Elena Sánchez Martín-Fontecha[2], Aashima Khosla[1], Alexandra R.F. White[1], Sunhyun Chang[1], Pilar Cubas[2], David C. Nelson[1C]

[1]Department of Botany and Plant Sciences, University of California, Riverside, CA 92521, United States of America

[2]Plant Molecular Genetics Department, Centro Nacional de Biotecnología/CSIC, Campus Universidad Autónoma de Madrid, Madrid, Spain

[C]Corresponding author, david.nelson@ucr.edu

This PDF file includes:

Supplemental information text

Supplemental figures 1 to 11

Supplemental table 1

Supplemental Information text

Extended Description of Materials and Methods

Plant growth conditions

Plants were grown under white light (MaxLite LED T8 4000K, ~110 µmol m^{-2} s^{-1}) with LD photoperiod (16 h light/8 h dark) at 21°C. Soil was supplemented with Gnatrol WDG and Marathon (imidacloprid). *Agrobacterium tumefaciens* (GV3101 pMP90)–mediated transformation of *Arabidopsis* was performed using the floral dip method as described previously (Clough and Bent, 1998). All characterized transgenic lines were homozygous.

Chemical compounds

KAR_1, KAR_2 and *rac*-GR24 were synthesized as previously reported (Goddard-Borger et al., 2007). $GR24^{5DS}$ and $GR24^{ent\text{-}5DS}$ enantiomers were purified from *rac*-GR24 by chiral-phase HPLC as described (Scaffidi et al., 2014). 10 mM or 50 mM stocks were prepared in acetone and stored at -20°C, and freshly diluted in aqueous solutions before use.

Hypocotyl assay

Hypocotyl growth under red light was performed as described previously, but in a HiPoint DCI-700 LED Z4 growth chamber (Nelson et al., 2011). Surface-sterilized seeds were plated on 0.5x Murashige-Skoog (MS) media with 1 µM KAR_2, 1 µM *rac*-GR24, 0.5 µM $GR24^{5DS}$, 0.5 µM $GR24^{ent\text{-}5DS}$ or 0.01% (v/v) acetone as mock control, stratified for 3 d at 4°C in darkness, treated with 3 h white light (~150 µmol $m^{-2}s^{-1}$) at 21°C, returned to darkness for 21 h at 21°C, and then grown under continuous red light (~30 µmol $m^{-2}s^{-1}$) at 21°C for 4 d before being photographed. Hypocotyl length was measured using ImageJ software (NIH).

Branching assay

The position of plants within flats was randomized to account for environmental variation. The number of primary rosette branches, not including the primary shoot, at least 1 cm in length was measured for each plant at global proliferative arrest (~7 weeks after germination).

RT-qPCR analysis

Total RNA was prepared and DNase-treated with the Spectrum Plant Total RNA Kit and On-Column DNase I Digestion Set (Sigma-Aldrich) from non-elongated axillary buds collected 10 d after anthesis or 7-d-old seedlings. First-strand cDNA was synthesized from 2 µg of total RNA with the iScript cDNA Synthesis Kit (Bio-Rad). Quantitative PCR was performed in a CFX384 Real-Time PCR Detection System (Bio-Rad) using Luna Universal qPCR Master Mix (NEB) with the following program: 5 min at 95°C and 45 cycles of 20 s at 95°C, 20 s at 60°C, and 20 s at 72°C, followed by melt curve analysis to analyze product specificity. The relative gene expression was calculated by $2^{\Delta Cq}$ [$\Delta C_q = C_q(\text{reference}) - C_q(\text{target})$]. Primers for *BRC1*, *RD29A*, *Cor15A*, *PKS5* and the *CACS* reference gene are previously described (Fujii et al. 2011; Wang et al. 2014; Soundappan et al., 2015).

Yeast Two-Hybrid Assay

To construct plasmids for yeast two-hybrid assays, the coding sequences for *D14*, *d14*^{seto}, and *d14*^{S97A} were cloned into pDONR221 Gateway entry vector by BP clonase reaction, sequence-verified, and recombined by LR clonase reaction into Gateway yeast expression vector pDest-GBKT7 to generate BD-D14, BD-d14^{seto}, and BD-d14^{S97A}, respectively. To make GAL4 DNA activation domain (AD) constructs, the coding sequences for *SMAX1*, *SMXL2*, *SMXL7*, *SMAX1*$_{D1M}$ and *SMXL7*$_{D1M}$ were cloned into pDONR221, sequence-verified, and moved into Gateway yeast expression vector pDest-GADT7. Direct interaction of two proteins was investigated by co-transformation of the respective plasmids in the yeast strain Y2HGold (Clontech) by the standard

lithium acetate-mediated method (Gietz and Woods, 2002). The transformed yeast strains were plated on SD/-Leu-Trp medium (Clontech) at 30°C for 3 d. Interactions in yeast were tested on SD/-Leu-Trp-His and SD/-Leu-Trp-His-Ade (Clontech) medium supplemented with 2 μM $GR24^{5DS}$ or 0.02% (v/v) acetone.

Transient Expression in *Nicotiana benthamiana*

N. benthamiana plants (3 weeks old) were used to express the various construct combinations by Agrobacterium (GV3101 pMP90)–mediated transient transformation of lower epidermal leaf cells as described previously (Khosla et al., 2020a).

FRET-ABP assay

N. benthamiana leaves were sprayed with 10 μM estradiol 24 hours after infiltration of Agrobacterium to induce protein expression from *LexA*:*SMXL7-mCherry* (pAB118), *LexA*:*SMAX1-mCherry* (pAB118), *LexA*:*SMXL7-mCherry-GFP* (pAB117) or *LexA*:*SMAX1-mCherry-GFP* (pAB117) (Bleckmann et al., 2010). *D14* and $d14^{seto}$ were cloned into binary vector pGWB505 and expressed under control of the 35S promoter in *N. benthamiana* leaves. The assay was performed 24 h after induction on a Leica TCS SP5 laser scanning confocal microscope with a 63 x/ 1.2 NA water immersion objective. The FRET-APB wizard of LAS-AF was used with the following parameters: acquisition speed 700 Hz; pinhole 60.7 μm; image format 512 x 512 pixels; zoom 6X. Regions Of Interest (ROIs) of 6 x 3.5 μm were photobleached with 10 repeated exposures (laser 561 nm, 100% power level). Images were processed using Leica the Application Suite Advanced Fluorescence software (LAS-AF). FRET Efficiency (E_{FRET}%) was measured as the increase of donor fluorescence (GFP) intensity after photobleaching of the acceptor (mCherry). $E_{FRET}\% = 100 * (D_{post} - D_{pre})/ D_{post}$. D_{pre} and D_{post} were the fluorescence intensity of the donor before and after photobleaching, respectively, which were quantified using a secondary ROI inside the bleached region of the bleached region of the first ROI.

Degradation assays in *N. benthamiana*

To generate ratiometric reporter constructs for degradation assays in *N. benthamiana*, *D14*, *d14*^{seto}, and *d14*^{S97A}, *SMAX1, SMXL2*, and *SMAX1*$_{D2}$ entry clones were transferred into the pRATIO1212 destination vector by Gateway LR reaction (Khosla et al., 2020a). To examine the time course of degradation, the wells of a black 96-well polystyrene plate (Corning Costar®) were filled with 200 µl chemical treatments [10 µM KAR_1, 10 µM GR24^{5DS}, 10 µM GR24$^{ent\text{-}5DS}$, or 0.02% (v/v) acetone]. Leaf discs were excised 3 d post-infiltration and transferred to the treatment plate (one leaf disc per well) with the abaxial side up. Relative fluorescence was measured in a CLARIOstar plate reader (BMG Labtech) in plate mode (slow kinetics) at the indicated time points with the following settings: spiral scan option; scan diameter (mm), 5; and number of flashes per well per cycle, 36. Optimal settings for fluorescence measurements of the mScarlet-I reporter (ex. 560-10 nm, em. 595-10 nm) and Venus reference (ex. 497-15 nm, em. 540-20 nm) proteins were described previously (Khosla et al., 2020a). Degradation was quantified as mScarlet-I/Venus fluorescence intensity ratios after subtracting background fluorescence signals measured in leaf discs transformed with RNA silencing suppressor P19.

Degradation assays in *Arabidopsis thaliana*

4-day-old *35S*:*D14-GFP and 35S*:*d14*^{seto}*-GFP* transgenic plants (Col-0 background) grown vertically were placed in a multiwell slide (µ-Slide, 8 wells IbiTreat, IBIDI) and immobilized with 200 µl of MS agar medium [0.5% (w/v) low melting point agar]. The *rac*-GR24 was supplemented to a final concentration of 5 µM. Equivalent volume of acetone was added for mock control. 3 Z-series section images were captured every 10 min for 16 h at 22 ºC with a Microfluor Leica DMI6000B fluorescence microscope using a 10x objective and 470 nm light to detect GFP. GFP signal was quantified with Fiji using Region of Interest (ROI) Multi Measure plugging after determining a threshold range to eliminate the background. The GFP signal variation of each plant over time was calculated as a percentage to the signal at t_0 when SL was just applied. Then we

obtained the relative GFP signal by normalizing the GFP signal to the mean of its mock control at each time point.

To monitor $SMAX1_{D2}$, SMXL7 or D14 degradation, 9-day-old plants expressing *UBQ*:*$SMAX1_{D2}$-LUC* (pRATIO2251) (Khosla et al., 2020b), *UBQ*:*SMXL7-LUC* (pB7M34GW) or *UBQ*:*D14-LUC* (pB7M34GW) grown on a white 96-well plate (Perkin Elmer OptiPlate 96) containing 200 µl 0.5x MS agar medium were sprayed with 2 mM D-luciferin and incubated 3 h before treatment to equilibrate. 5 µM $GR24^{5DS}$, 5 µM KAR_2, 300 mM mannitol, or corresponding solvent control were then sprayed along with 2 mM D-luciferin. Luciferase signal was measured using a CLARIOstar plate reader (BMG Labtech) under controlled 21ºC temperature. The luciferase activity (LA) of each plant at each time point was calculated relative to time zero as LA (%) = $[(cps_{tn} * 100) / cps_{t0}]$. Then we obtained the Relative Luciferase Activity (RLA) by normalizing LA of each treatment/genotype to the average of corresponding solvent control at each time point.

Osmotic stress tolerance assays

7-day-old seedlings grown on 0.5x MS agar were transplanted to 0.5x MS agar containing 300 mM mannitol to induce osmotic stress. Seedlings were photographed and assayed after 14 d mannitol treatment. The aerial parts of 3-5 seedlings were excised and weighed. Tissues were ground by a bead mill and homogenized in extract solution (95% ethanol + 5% water) at 4 °C overnight until bleached. The total chlorophyll content was quantified by measuring the absorbance of the supernatant at 647 and 665 nm and using the formula total Chl = $(17.90 * A_{647} + 8.08 * A_{665})$/mg fresh weight (Chen et al., 2013).

Statistical analysis

Data were analyzed by using JMP Pro 13 and Excel. For multiple comparisons of means, one-way ANOVA was performed followed by Tukey's HSD test ($p < 0.05$) or Student's t-test ($p < 0.05$). Two-sided Student's t-test was conducted for comparisons of

means between two groups. Graphs were produced using Prism v7 (GraphPad Software). Box plots show the median, 25th percentile, and 75th percentile. Whiskers indicate the minimum and maximum of the data range, and individual data points are overlaid. For sample sizes with $n \leq 4$, individual data points and the mean value are shown.

SI References

Bleckmann, A., Weidtkamp-Peters, S., Seidel, C. A. M., and Simon, R. (2010). Stem cell signaling in Arabidopsis requires CRN to localize CLV2 to the plasma membrane. *Plant Physiol.* **152**:166–176.

Chen, L.-J., Wuriyanghan, H., Zhang, Y.-Q., Duan, K.-X., Chen, H.-W., Li, Q.-T., Lu, X., He, S.-J., Ma, B., Zhang, W.-K., et al. (2013). An S-domain receptor-like kinase, OsSIK2, confers abiotic stress tolerance and delays dark-induced leaf senescence in rice. *Plant Physiol.* **163**:1752–1765.

Clough, S. J., and Bent, A. F. (1998). Floral dip: a simplified method for Agrobacterium-mediated transformation of Arabidopsis thaliana. *Plant J.* **16**:735–743.

Fujii, H., Verslues, P. E., and Zhu, J.-K. (2011). Arabidopsis decuple mutant reveals the importance of SnRK2 kinases in osmotic stress responses in vivo. *Proc. Natl. Acad. Sci. U. S. A.* **108**:1717–1722.

Khosla, A., Rodriguez-Furlan, C., Kapoor, S., Van Norman, J. M., and Nelson, D. C. (2020a). A series of dual-reporter vectors for ratiometric analysis of protein abundance in plants. *Plant Direct* **4**.

Khosla, A., Morffy, N., Li, Q., Faure, L., Chang, S. H., Yao, J., Zheng, J., Cai, M. L., Stanga, J., Flematti, G. R., et al. (2020b). Structure–Function Analysis of SMAX1 Reveals Domains That Mediate Its Karrikin-Induced Proteolysis and Interaction with the Receptor KAI2. Plant Cell 32:2639–2659.

Scaffidi, A., Waters, M. T., Sun, Y. K., Skelton, B. W., Dixon, K. W., Ghisalberti, E. L., Flematti, G. R., and Smith, S. M. (2014). Strigolactone Hormones and Their Stereoisomers Signal through Two Related Receptor Proteins to Induce Different Physiological Responses in Arabidopsis. *Plant Physiol.* **165**:1221–1232.

Soundappan, I., Bennett, T., Morffy, N., Liang, Y., Stanga, J. P., Abbas, A., Leyser, O., and Nelson, D. C. (2015). SMAX1-LIKE/D53 Family Members Enable Distinct MAX2-Dependent Responses to Strigolactones and Karrikins in Arabidopsis. *Plant Cell* **27**:3143–3159.

Wang, X.-H., Li, Q.-T., Chen, H.-W., Zhang, W.-K., Ma, B., Chen, S.-Y., and Zhang, J.-S. (2014). Trihelix transcription factor GT-4 mediates salt tolerance via interaction with TEM2 in Arabidopsis. *BMC Plant Biol.* **14**:339.

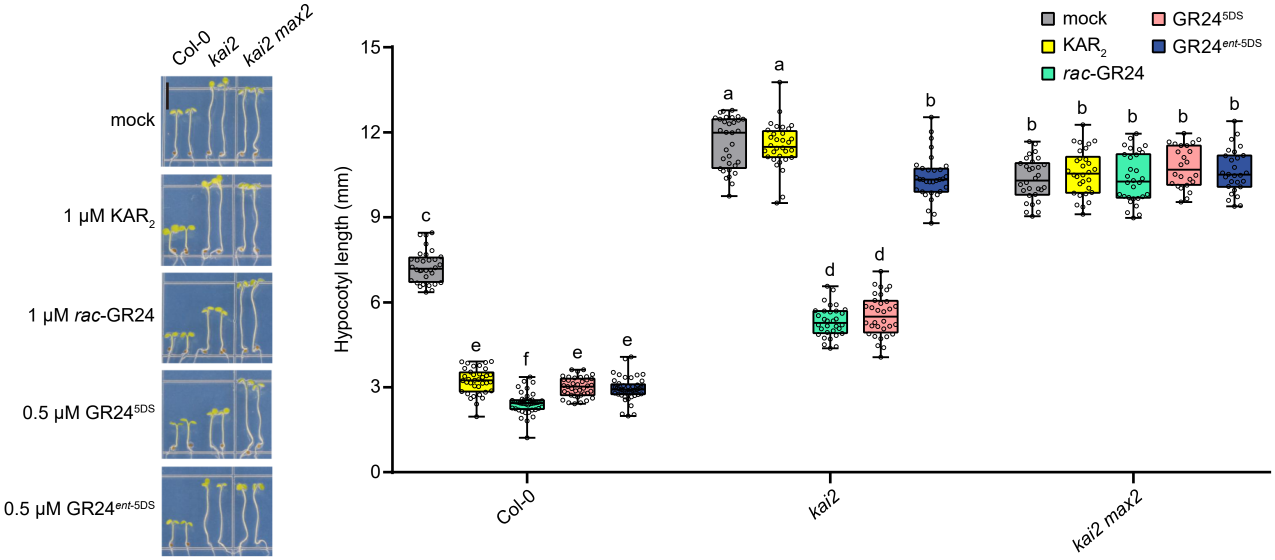

Supplemental Figure 1. Hypocotyl elongation of *kai2* is inhibited by *rac*-GR24 and $GR24^{5DS}$ through *MAX2*.
Hypocotyl lengths of 5-d-old seedlings of Col-0 (wild type), *kai2*, and *kai2 max2* are grown under continuous red light for 4 d on the 0.5x MS agar media containing 1 μM KAR_2, 1 μM *rac*-GR24, 0.5 μM $GR24^{5DS}$, 0.5 μM $GR24^{ent\text{-}5DS}$ or acetone. Bar = 5 mm. Box-and-whisker plots with the same letter are not significantly different from one another (Tukey HSD, $p < 0.05$, $n \geq 30$).

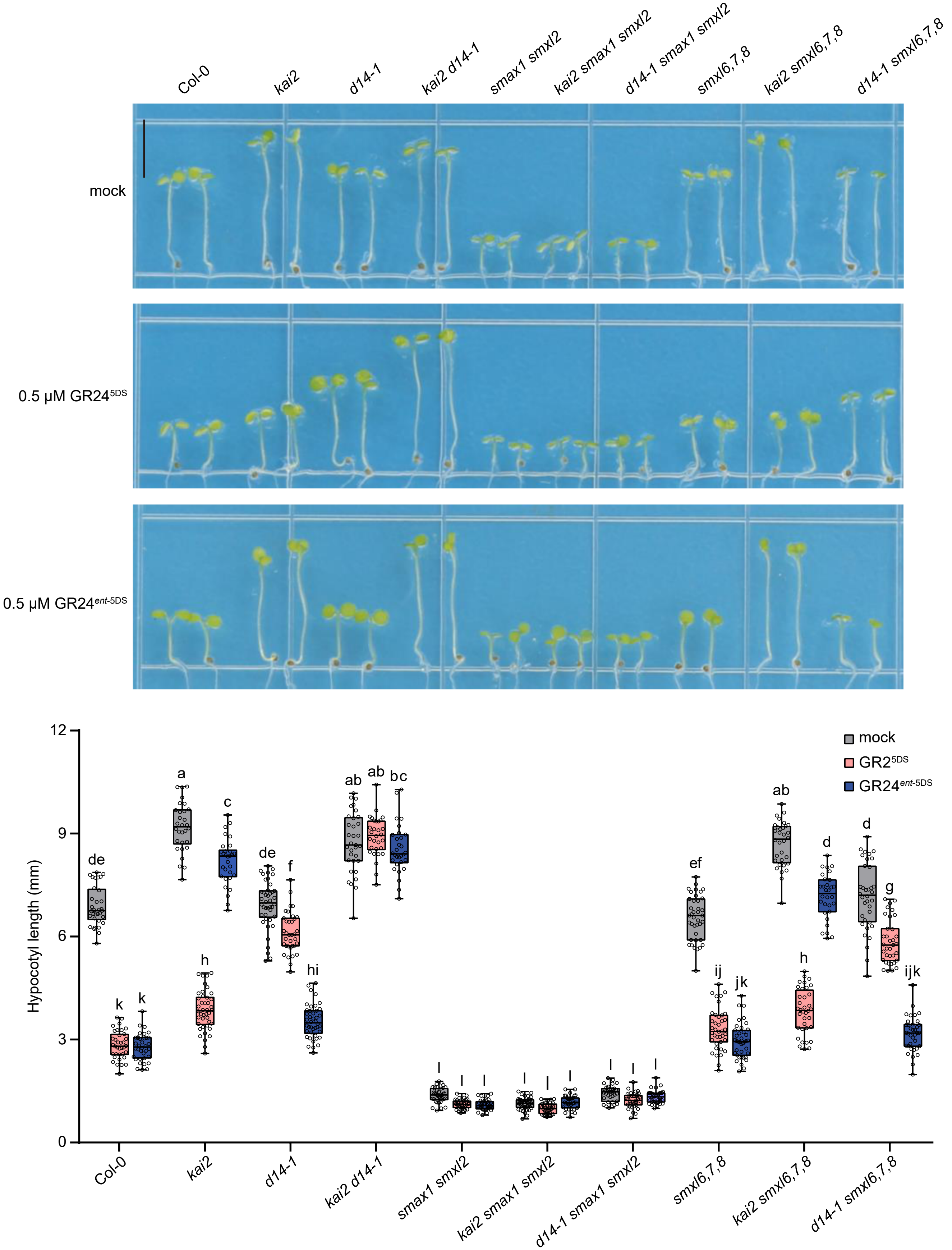

Supplemental Figure 2. Hypocotyl growth of plant materials in Figure 1B with GR24^{5DS} or GR24$^{ent\text{-}5DS}$ treatment. Hypocotyl lengths of 5-d-old seedlings of Col-0, *kai2*, *d14-1*, *kai2 d14-1*, *smax1 smxl2*, *kai2 smax1 smxl2*, *d14-1 smax1 smxl2*, *smxl6,7,8*, *kai2 smxl6,7,8* and *d14-1 smxl6,7,8* are grown under continuous red light for 4 d on the 0.5x MS agar media containing 0.5 µM GR24^{5DS}, 0.5 µM GR24$^{ent\text{-}5DS}$ or acetone. Mock-treated seedling data are duplicated in Figure 1, which shows additional data from this experiment. Bar = 5 mm. Box-and-whisker plots with the same letter are not significantly different from one another (Tukey HSD, $p < 0.05$, $n \geq 30$).

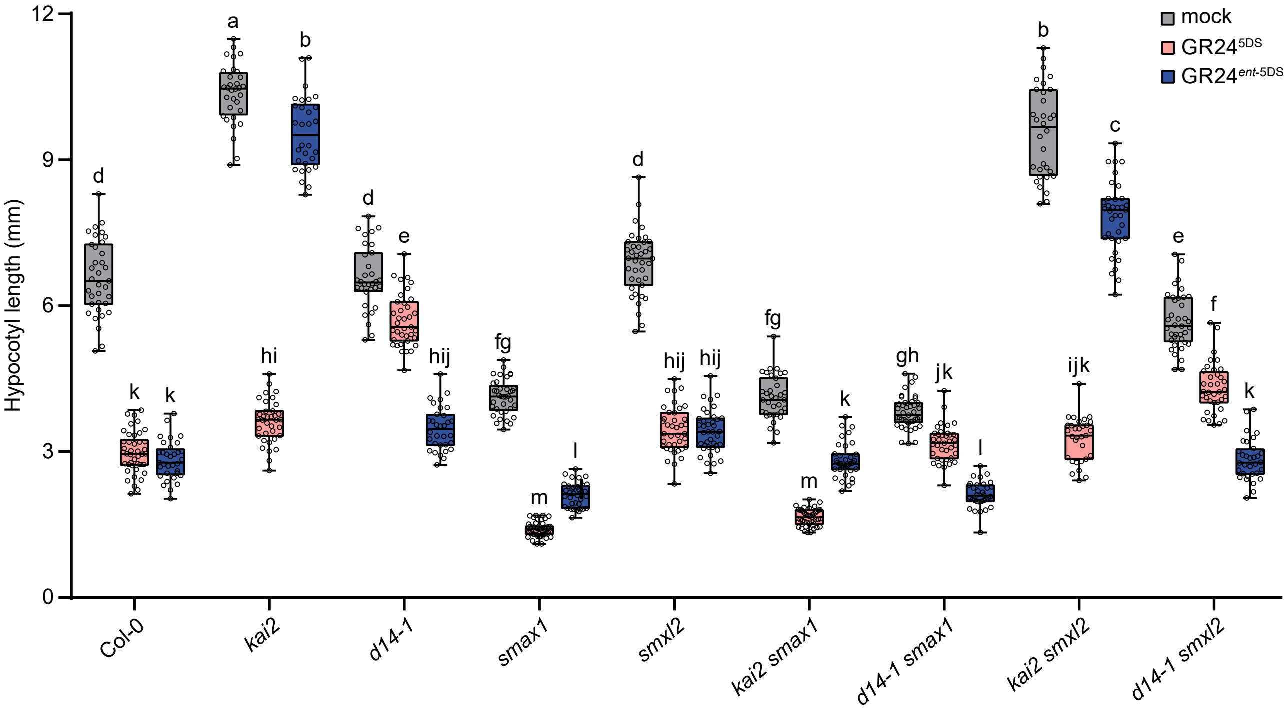

Supplemental Figure 3. Hypocotyl growth of plant materials in Figure 1C with GR24^{5DS} or GR24$^{ent\text{-}5DS}$ treatment. Hypocotyl lengths of 5-d-old seedlings of Col-0, *kai2*, *d14-1, smax1, smxl2, kai2 smax1, d14-1 smax1, kai2 smxl2, d14-1 smxl2* are grown under continuous red light for 4 d on the 0.5x MS agar media containing 0.5 µM GR24^{5DS}, 0.5 µM GR24$^{ent\text{-}5DS}$ or acetone. Mock-treated seedling data are duplicated in Figure 1C, which shows additional data from this experiment. Box-and-whisker plots with the same letter are not significantly different from one another (Tukey HSD, $p < 0.05$, $n \geq 30$).

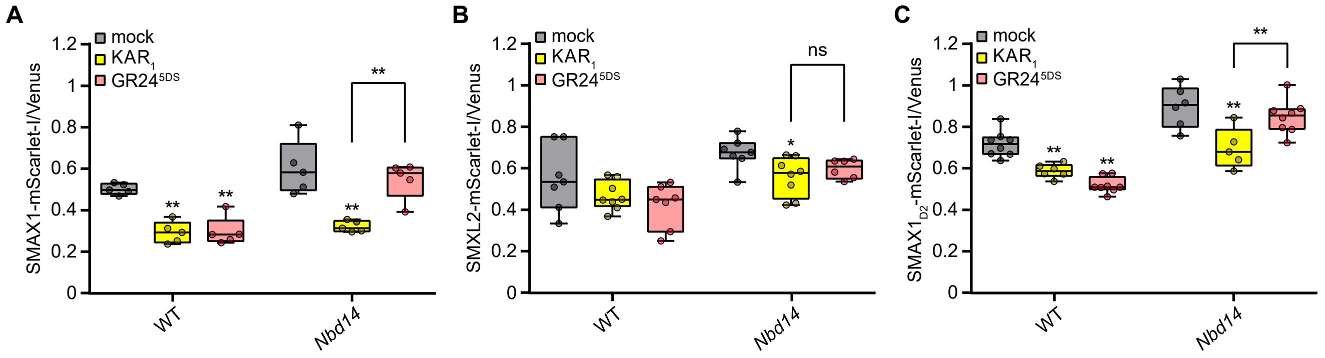

Supplemental Figure 4. Degradation of SMAX1, SMXL2 or SMAX1$_{D2}$ after 4 h treatment of KAR$_1$ or GR24^{5DS}. Relative fluorescence from the SMAX1-mScarlet-I reporter **(A)** or SMXL2-mScarlet-I reporter **(B)** or SMAX1$_{D2}$-mScarlet-I reporter **(C)** and the Venus reference after transient expression of the ratiometric system in wt tobacco and *Nbd14* is shown. Leaf discs are treated with acetone, 10 μM KAR$_1$, or 10 μM GR24^{5DS} for 4 h. Mock-treated seedling data are duplicated in Figure 2A to 2C, which show additional data from this experiment. n = 5-8 leaf discs. ns indicates no significance. *$p < 0.05$, **$p < 0.01$, Student's t-test comparisons to the relative fluorescence at 0 h or between compared pairs.

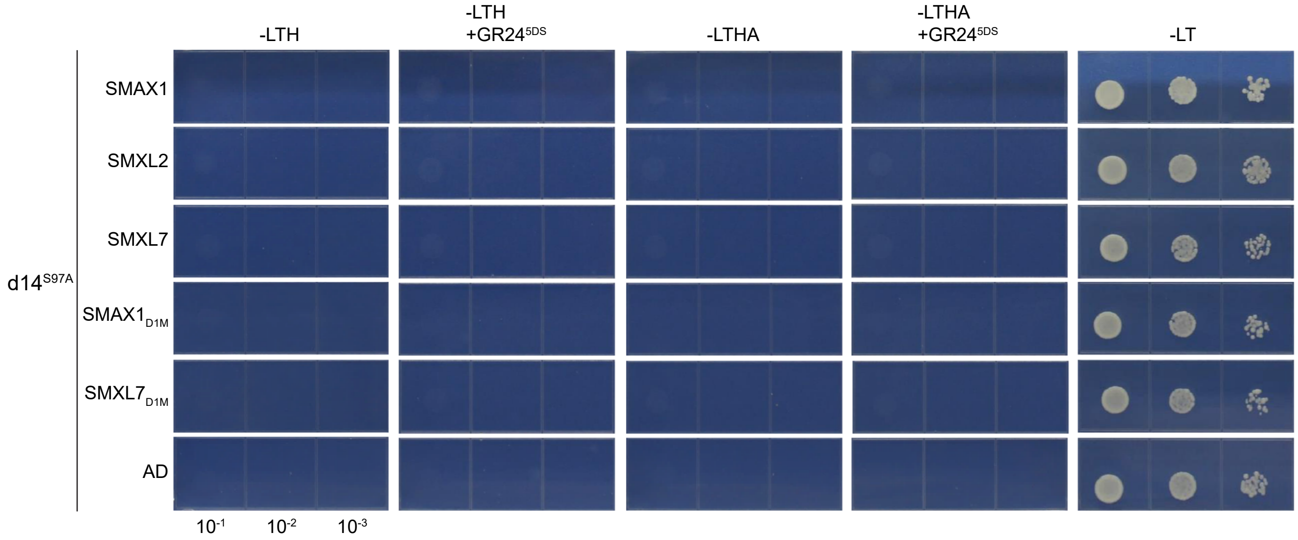

Supplemental Figure 5. Yeast two-hybrid assays for d14^{S97A} interactions with SMAX1, SMXL2, SMXL7, SMAX1$_{D1M}$ and SMXL7$_{D1M}$.

The d14^{S97A} is fused to GAL4-BD. SMAX1, SMXL7 and their D1M domains are fused to GAL4-AD. Serial 10-fold dilutions of yeast cultures are spotted onto selective growth medium (-L, -Leu; -T, -Trp; -H, -His; -A, -Ade) that is supplemented with 2 µM GR24^{5DS} or acetone.

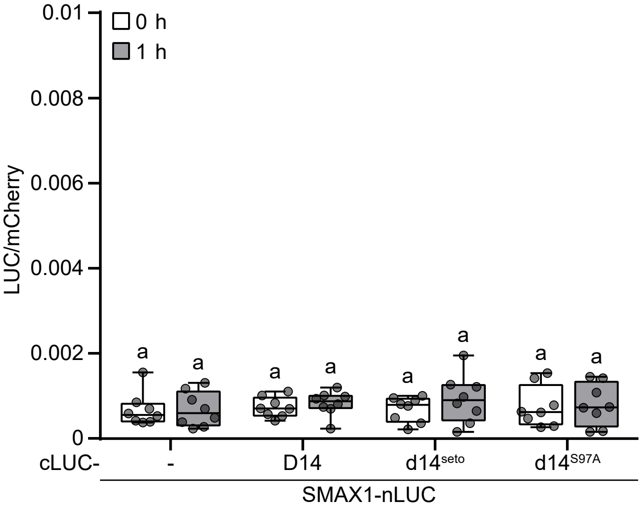

Supplemental Figure 6. D14, d14^{seto} and d14^{S97A} interactions with SMAX1 in split-luciferase assay.
N. benthamiana leaves are transiently co-transformed with *Agrobacterium tumefaciens* strains carrying cLUC, nLUC, or indicated fusions as well as a strain carrying an mCherry transgene as a transformation control. Luminescence is measured before and 1 h after treatment with 10 μM GR24^{5DS}, and normalized against mCherry fluorescence. Box-and-whisker plots with the same letter are not significantly different from one another (Student's t-test, $p < 0.05$, $n = 8$ leaf discs).

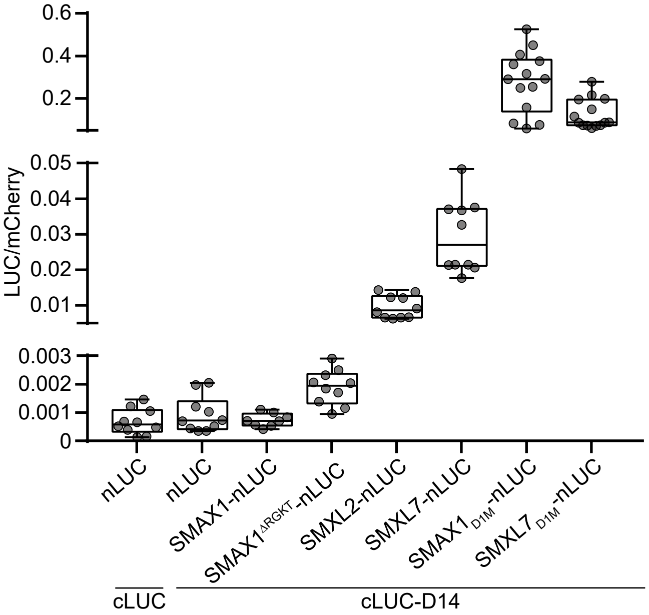

Supplemental Figure 7. Baseline of D14 interactions with SMAX1, SMAX1$^{\Delta RGKT}$, SMXL2, SMXL7, SMAX1$_{D1M}$ and SMXL7$_{D1M}$ in split-luciferase assay.

N. benthamiana leaves are transiently co-transformed with *Agrobacterium tumefaciens* strains carrying cLUC, nLUC, or indicated fusions as well as a strain carrying an mCherry transgene as a transformation control. Luminescence is measured before 10 μM GR24^{5DS} treatment, and normalized against mCherry fluorescence. n = 7-15 leaf discs. The data are duplicated in Figure 3B to 3F and Supplemental Figure 6, which show additional data from this experiment.

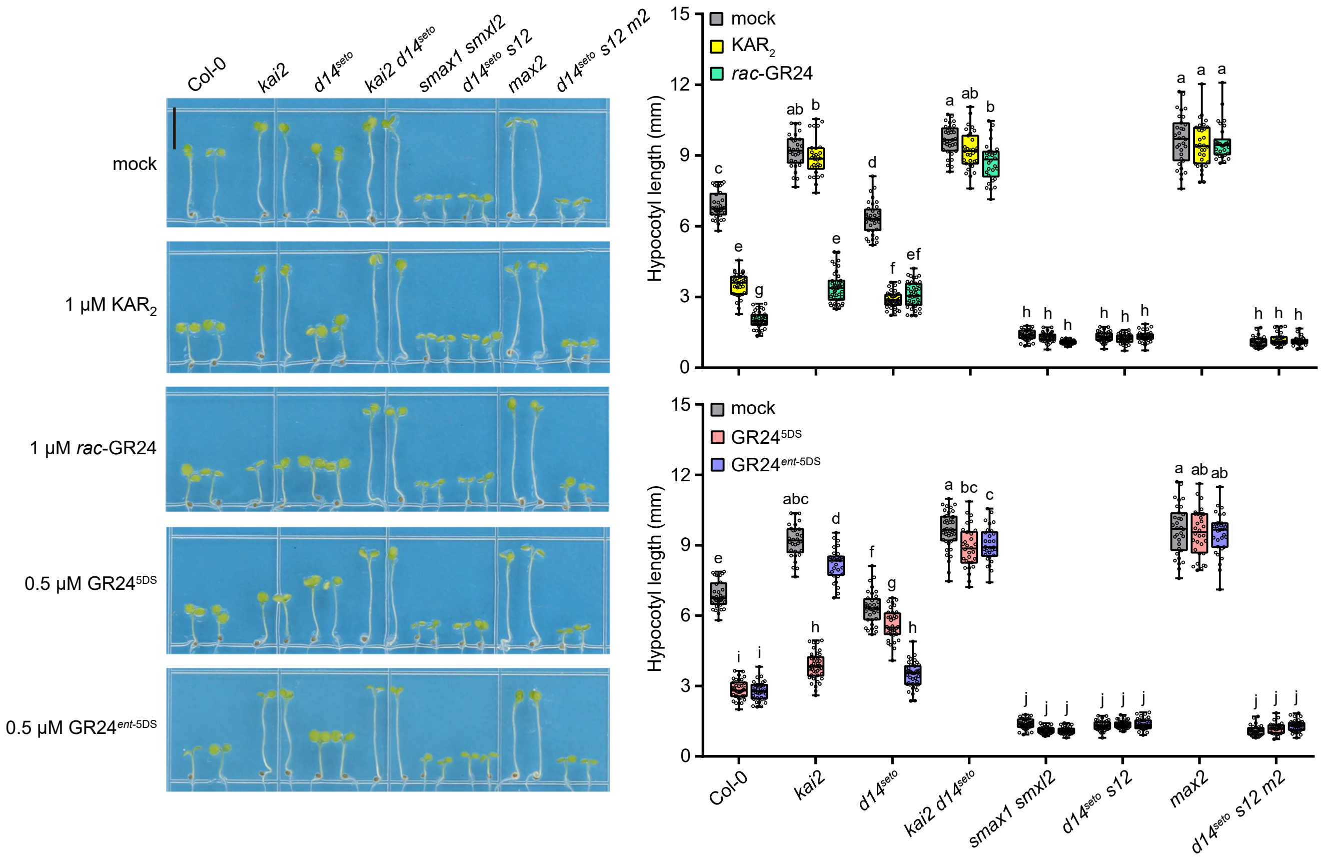

Supplemental Figure 8. Hypocotyl growth of Col-0, *kai2*, *d14*^{seto}, *kai2 d14*^{seto}, *smax1 smxl2*, *d14*^{seto} *smax1 smxl2*, *max2*, *d14*^{seto} *smax1 smxl2 max2* seedlings under different treatments.

Plants are grown under continuous red light for 4 d on the 0.5x MS agar media containing 1 µM KAR_2, 1 µM *rac*-GR24, 0.5 µM GR24^{5DS}, 0.5 µM GR24$^{ent\text{-}5DS}$ or acetone. Bar = 5 mm. Box-and-whisker plots with the same letter are not significantly different from one another (Tukey HSD, $p < 0.05$, $n \geq 30$).

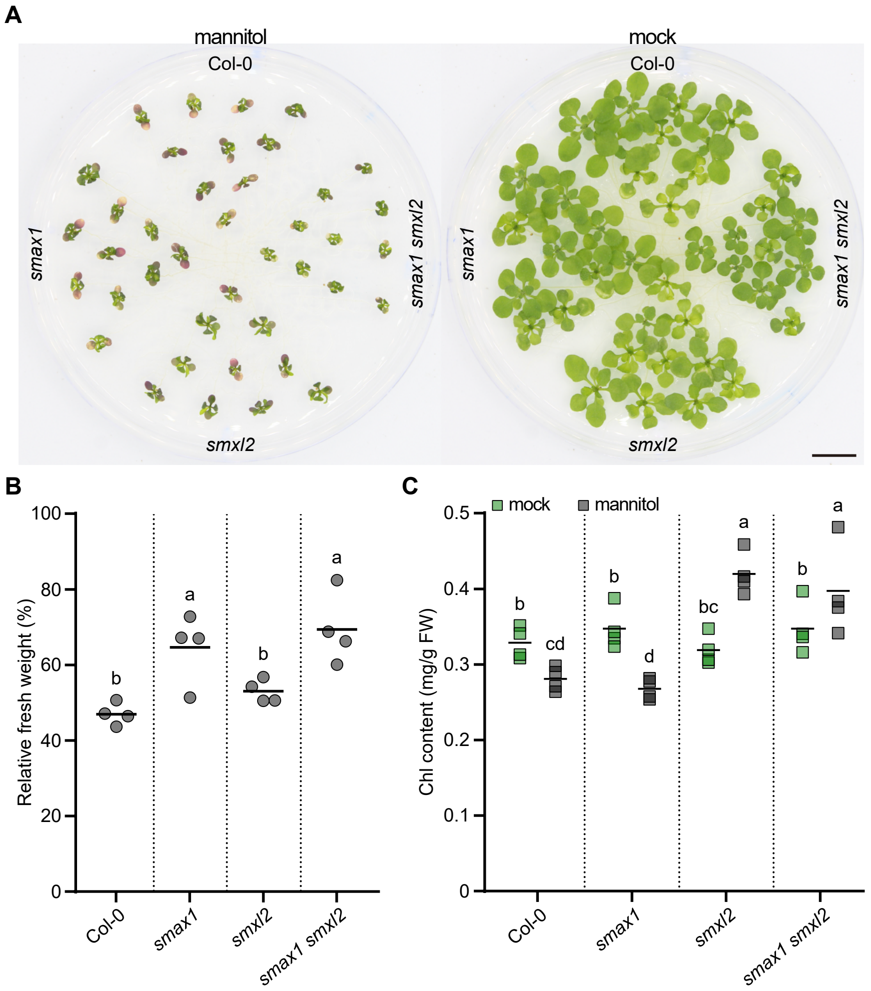

Supplemental Figure 9. Osmotic stress tolerance of Col-0, *smax1*, *smxl2* and *smax1 smxl2*.
(A) 21-day-old seedlings of Col-0, *smax1*, *smxl2* and *smax1 smxl2* grown in mock or 300 mM mannitol condition for 14 days. Bar = 1 cm.
(B) Relative fresh weights of plant materials used in **(A)** to application of 300 mM mannitol. The weights of aerial parts from plants grown on 0.5x MS agar medium containing 300 mM mannitol are scaled to that from plants grown on 0.5x MS agar medium. Scatter dot plots with the same letter are not significantly different from one another (bar indicates mean; n = 4; Student's t-test, $p < 0.05$).
(C) Chlorophyll (Chl) contents in the aerial parts of Arabidopsis seedlings used in **(A)**. Others are as in **(B)**.

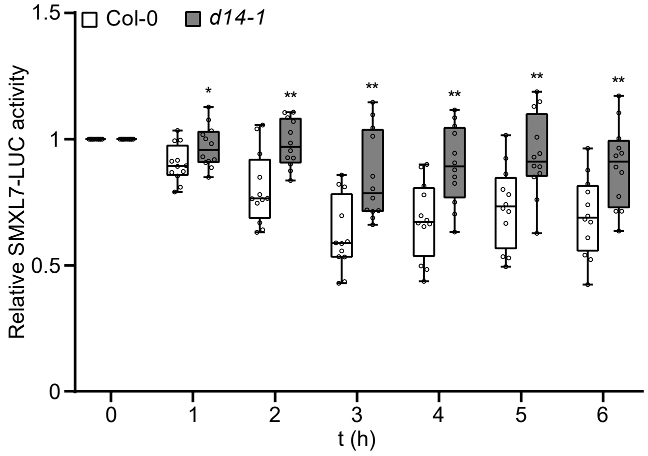

Supplemental Figure 10. Osmotic stress triggers the SMXL7 degradation.
Bioluminescence of SMXL7-LUC in Col-0 and *d14-1* backgrounds. Seedlings were treated with 300 mM mannitol or water control. Bioluminescence is shown as relative LUC activity and is monitored for 6 h after treatment. n = 12 seedlings. $*p < 0.05$, $**p < 0.01$, Student's t-test comparisons to Col-0 control at each time point.

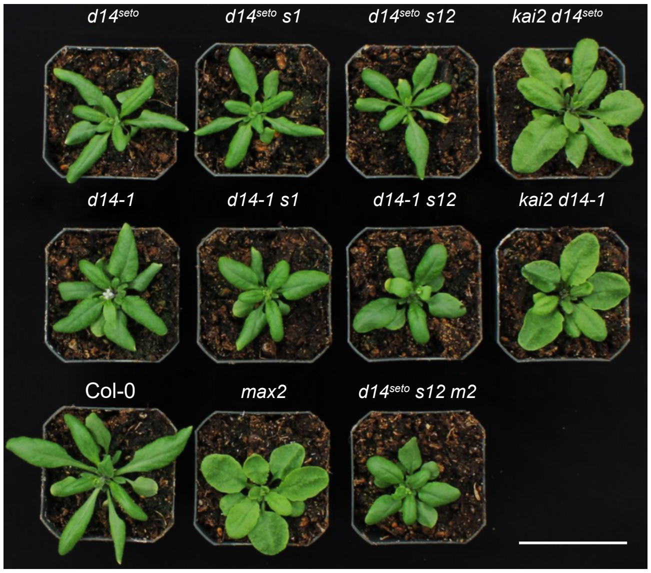

Supplemental Figure 11. Rosette phenotypes of plant materials in Figure 4A.
Col-0, *d14-1*, *kai2 d14-1*, *d14-1 smax1*, *d14-1 smax1 smxl2*, $d14^{seto}$, *kai2* $d14^{seto}$, $d14^{seto}$ *smax1*, $d14^{seto}$ *smax1 smxl2*, *max2* and $d14^{seto}$ *smax1 smxl2 max2* plants are grown for 4 weeks under a long-day photoperiod (16 h light/8 h dark) before imaging. Bar = 5 cm.

Supplemental Table 1. Primers used in this study.

Primer Name	Primers Sequences
**Primers for genotyping**	
kai2-F	CACTTGGTTCCACATCTGGTC
kai2-R	GAGATTTGAGTAACGATCGAAGTCG
max2-1_dCAPS-F	TGTCCGAATTTGGAAGAGATTAGG
max2-1_dCAPS-R	CAAGAAGAATCTTTCCCATAAACTCGAAT
d14-1-WiscLoxHS-LP	AAGAATATGGCAAGTGCAAC
d14-1-WiscLoxHS-RP	GATGATTCCGATCATAGCG
L4_WiscLoxHS	TGATCCATGTAGATTTCCCGGACATGAAG
smax1-2-Salk-LP	GTGGCAACTGTTTAGGCTGAG
smax1-2-Salk-RP	AAGCTAGCTTTTCAAGTCCCG
smxl6-4-Salk-LP	AGCCAGAGAAAGACTCGAACC
smxl6-4-Salk-RP	TCCGAAATTAAGCTCGATGTG
smxl8-1-Salk-LP	GAATCACAAATTCTGCATGGC
smxl8-1-Salk-RP	CTGACGAAGCTCCACTTTCAC
Salk-LBb1.3	ATTTTGCCGATTTCGGAAC
smxl7-3-WiscDsLox-LP	GATCAAGAAACGAACGCTGAG
smxl7-3-WiscDsLox-RP	CGTATTAGCCTCTCGGATTCC
WiscDsLox-LB-p745	AACGTCCGCAATGTGTTATTAAGTTGTC
smxl2-1-Sail-LP	TGACATACACCGATCACCAC
smxl2-1-Sail-RP	GTATCATCATCCCACTTTGCATAC
Sail-LB1	GCCTTTTCAGAAATGGATAAATAGC
seto5_dCAPS-F	GGAGGATTCGAAGAAGGTGAGATTG
seto5_dCAPS-R	CGTACGCATATTAAACAAAGTACGGC
**Primers for constructs**	
D14-cLUC-F	TCGTACGCGTCCCGGGGCATGAGTCAACACAACATCTTAGAA
D14-cLUC-R	CGAACGAAAGCTCTGCAGTCACCGAGGAAGAGCTCGCC
SMAX1-nLUC-F	AACACGGGGGACGAGCTCATGAGAGCTGGTTTAAGTACGAT
SMAX1-nLUC-R	GGACGCGTACGAGATCTGTACTGCCAAAGTAATAGTTGTCG
SMXL2-nLUC-F	GAGAGAACACGGGGGACGAGCTCATGAGAGCAGATTTGATTACTATACAGC
SMXL2-nLUC-R	CCGGGACGCGTACGAGATCTG AACGACCACCGTCCTGATACTAC
SMXL7-nLUC-F	AACACGGGGGACGAGCTCATGCCGACACCAGTAACCACG
SMXL7-nLUC-R	GGACGCGTACGAGATCTGGATCACTTCGACTCTCGCCGG
$SMAX1_{D1M}$-nLUC-F	GAGAGAACACGGGGGACGAGCTCATGTTACAACAGAACGCTTCGTC
$SMAX1_{D1M}$-nLUC-R	CCGGGACGCGTACGAGATCTGGATGTTATTATTGTTCTGCACTGATTCAG
$SMXL7_{D1M}$-nLUC-F	GAGAGAACACGGGGGACGAGCTCATGGACATTAAACTCGACGTGCTTCATC
$SMXL7_{D1M}$-nLUC-R	CCGGGACGCGTACGAGATCTGTGGTTCTTGGTTCTTCGATGCGTAG
**Primers for qRT-PCR**	
BRC1-F	TCGCGACAACCCTTTCTCACCAT
BRC1-R	CGGTCGTGTTAGTATTGCTGCCTCT
RD29A-F	GCCGACGGGATTTGACG
RD29A-R	GCCGACGGGATTTGACG
Cor15A-F	ATGGCGATGTCTTTCTCAGGAGCTGTT
Cor15A-R	TTTTATCCGTCACGAAATCTGAAGCTT

PKS5-F	GTTTGCGAGAGAGGAGAATCTG
PKS5-R	CCACAAGCAAATCATTCAACCG
CACS-F	GGAGAAGAGAGGGCCTTGCTTACAA
CACS-R	TTAGCTGGGCGAGATTTCATTTCTG
